# Cross-species analysis of abiotic stress in hydroponic leafy crops reveals conserved regulatory networks and key divergences

**DOI:** 10.3389/fpls.2025.1613016

**Published:** 2025-07-07

**Authors:** Jia Min Lee, Jong Ching Goh, Eugene Koh, Daniela Mutwil-Anderwald, Marek Mutwil

**Affiliations:** ^1^ School of Biological Sciences, Nanyang Technological University, Singapore, Singapore; ^2^ Department of Plant and Environmental Sciences, University of Copenhagen, Copenhagen, Denmark

**Keywords:** abiotic, stress, transcriptomic, hydroponic, comparative

## Abstract

Hydroponics is emerging as a vital method for producing resilient leafy greens in controlled environments. To systematically capture how hydroponically grown crops respond to stress, we subjected three species—cai xin, lettuce, and spinach—to 24 environmental and nutrient treatments. Growth measurements showed that extreme temperatures, reduced photoperiods, and severe macronutrient (N, P, K) deficiencies significantly limit fresh weight. Transcriptomic profiling (276 RNA-seq libraries) highlighted strong, shared downregulation of photosynthesis-related genes and upregulation of stress response and signaling genes across all three species. Leveraging a novel pipeline that merges regression-based gene network inference with orthology, we identified highly conserved gene regulatory networks (GRNs) spanning all three species—marking the first cross-species analysis of stress-responsive GRNs in economically important hydroponic leafy vegetables. These networks are anchored by well-known transcription factor families (e.g., WRKY, AP2/ERF, GARP), yet show lineage-specific differences compared to Arabidopsis, suggesting partial divergence in key regulatory components. Lastly, we introduce StressCoNekT (https://stress.plant.tools/), an interactive, publicly available database that hosts our transcriptomic data and offers comparative tools to accelerate the discovery of robust stress-responsive genes and cross-species analysis. This study not only deepens our understanding of abiotic stress adaptation in hydroponic systems but also provides a critical foundation for breeding stress-resilient crops and developing smart agriculture solutions.

## Introduction

With changing dynamics in global food markets and an expanding population, more studies are needed to develop resilient food production systems in urban environments. Hydroponics has emerged as a potential solution for urban farming (Martin and Molin, 2019), as this technology can be deployed on rooftops and indoors, and allows controlled light, temperature and nutrient levels to maintain high growth rates, with the additional advantage of saving water. Thus, in light of food security, independence from soil quality and local climate, hydroponic techniques have become a major part of global agriculture, particularly for leafy greens (Rajaseger et al., 2023). In combination with ‘smart farming,’ which uses sensors and other control systems to constantly monitor nutrient levels and plant vitality, hydroponics can produce up to 20 times the yield per acre of soil-planted crops—e.g., for lettuce—with only 1/20th the amount of water (Majid et al., 2021; Gul and Bora, 2023). Still, hydroponics also faces challenges including high energy demands, water-quality oversight, disease and pest management complexities, co-cultivation limitations, and the lack of crop varieties specifically bred for indoor cultivation.

Abiotic stresses can affect hydroponically grown plants, for example when nutrient levels are too high or too low, or when temperature and light conditions are not optimal for plant growth. High temperatures greatly reduce the efficiency of photosynthesis and respiration due to changes in membrane fluidity and permeability (Ding et al., 2020; Zhao et al., 2020). Heat also leads to an increase in ROS generation in the photosystems, which in turn induces lipid peroxidation, inactivates enzymes, and degrades proteins (Zhao et al., 2020). Low levels of macronutrients, such as nitrogen (N), phosphorus (P), and potassium (K), cause typical deficiency symptoms in plants (van Maarschalkerweerd and Husted, 2015). As an essential component of DNA, RNA, proteins, and chlorophyll, N deficiency causes drastic changes in plant morphology and metabolism, as reflected in stunted growth, small leaves, and chlorosis (de Bang et al., 2021). NO3^−^ also acts as a signaling molecule and plays a central role in protecting plants from various environmental stresses (Wang et al., 2018; Khan et al., 2023). Phosphorus is essential for nucleic acids (DNA, RNA), phospholipids in cell membranes, and is central to protein phosphorylation. Low P levels have a profound impact on energy metabolism (ATP, NADPH), and consequently on photosynthesis and respiration (de Bang et al., 2021). The physiological functions of K include stomatal regulation, photosynthesis, and water uptake (Johnson et al., 2022). Potassium deficiency activates a range of sensing and signaling systems in plants, involving ROS, Ca2^+^, phytohormones, and microRNAs (de Bang et al., 2021). Increasing light intensities within a physiological range (up to 300 uE) typically result in better growth and higher levels of soluble sugars and proteins in leafy vegetables. However, high light intensity can alter leaf morphology and lead to leaf curling and/or tipburn (Miao et al., 2023).

Another important consideration is how these stresses affect photosynthetic processes and carbon fixation. Stresses such as heat, cold, drought, and high light can accelerate photodegradative processes in the chloroplast, thereby reducing photosynthetic efficiency (Chauhan et al., 2023). Adaptive physiological changes, such as stomatal closure and leaf or chloroplast movement, aim to dynamically adjust the photosynthetic and carbon fixation rates, balancing growth and stress acclimation. These responses are typically regulated through broader stress signaling networks that protect the plant and enhance adaptation to future stress conditions (Murchie et al., 2022). Some of these networks, especially in the context of biotic stresses and herbivory, are conceptualized under the Growth-Defense Tradeoff (GDT), which posits that defense and growth signaling pathways antagonistically regulate one another (Huot et al., 2014). As previously discussed, nutrient stress leads to deficiencies in key intermediates involved in plant homeostasis, which can impair both the functioning of the photosynthetic machinery and growth signaling pathways (Murchie et al., 2022). Given the diverse genetic backgrounds of different plant species, sensitivity to abiotic stress and the associated gene regulatory programs are likely to vary; however, the extent of these differences remains underexplored across species.

Despite the increasing relevance of hydroponics for crop production, few studies have examined how different plant species respond to abiotic stress within this system. Most existing knowledge derives from model plants like *Arabidopsis thaliana* or staple crops such as rice, which are typically grown under soil-based conditions (Kobayashi and Nishizawa, 2012; Bouain et al., 2014; Bouain et al., 2018). Transcriptomics has been widely used to study stress (Hirayama and Shinozaki, 2010), yet insights into conserved stress responses remain limited. Plants have evolved mechanisms to perceive abiotic stress and adjust gene expression—along with growth and development—to ensure survival and reproduction (Gong et al., 2020). However, comparative transcriptomic studies—especially in hydroponically grown leafy vegetables—remain scarce, leaving significant gaps in our understanding of shared versus species-specific stress responses. In addition, inconsistencies in stress application, developmental stages, and experimental design in existing studies often hinder direct cross-species comparisons (Julca et al., 2023). To address these limitations, we conducted a systematic investigation of 24 different environmental and nutrient conditions affecting the growth yield of three hydroponically grown leafy crops: cai xin, lettuce, and spinach. Using a unified experimental framework, we identified growth conditions that either maximize yield or induce abiotic stress, enabling direct comparisons of gene expression responses across species. This design enabled us to examine the conservation of stress-responsive genes and regulatory modules, offering deeper insight into how plants perceive and transcriptionally adapt to stress in controlled-environment agriculture.

We observed that abiotic stresses significantly impact multiple biological pathways. We then conducted an in-depth, parallel gene expression analysis across the three species and identified sets of conserved, high-confidence genes likely involved in abiotic stress responses across the plant kingdom. To construct high-confidence gene regulatory networks for stress response, we developed a novel method integrating regression analysis with genomic data. Surprisingly, although the gene regulatory networks were largely conserved across the three crop species, comparison of key transcription factors to their *A. thaliana* counterparts revealed low functional conservation, suggesting substantial functional differences in transcription factor activity across species. Finally, we also established an online stress-response database of gene expression profiles for the three crops (https://stress.plant.tools/), enabling researchers to perform comparative analyses and facilitate the discovery of stress-responsive genes.

## Methods

### Growth conditions and chambers

We used Aspara^®^ Nature+ Smart Growers (Growgreen Ltd., Hong Kong), which were placed either in an MT-313 Plant Growth Chamber (HiPoint, Taiwan), a PGC-9 series controlled environment chamber (Percival Scientific, Inc., Perry, US), or under ambient conditions of 23°C–24°C for cai xin and lettuce, and 22°C (for spinach) across different laboratories.

### Germination of cai xin and lettuce

Two to three seeds were placed in each seed holder of the Aspara^®^ unit, which was filled with tap water and covered with a germination dome. Germination occurred under continuous white light for 24 h (40 μmol·m^−2^·s^−1^) at a temperature of 23°C–24°C. The Aspara^®^ Smart Grower Hydroponic System operates using an ebb-and-flow system and holds 2 L of medium.

### Germination of spinach (*Spinacia oleracea* var Carmel)

Spinach seeds were sown on cotton balls, kept in the dark, and regularly sprayed with water to maintain moisture. Within 3 days, 80% of the seedlings had germinated, indicated by visible radicles on the seed coat (designated as DAG 0, or days after germination). Germinated seedlings were exposed to continuous white light for 24 h (40 μmol·m^−2^·s^−1^) at a temperature of 23°C–24°C.

### Growth medium

The half-strength Hoagland’s solution consisted of KH_2_PO4 (500 μM), KNO_3_ (3,000 μM), Ca(NO_3_)_2_ × 4 H_2_O (2,000 μM), and MgSO_4_ × 7 H_2_O (1,000 μM). To prepare the solution, 0.5 mL of micronutrient stock (1,000×), was added to 1 L of the half-strength Hoagland’s solution. One liter of the micronutrient stock solution contained H_3_BO_3_ (2.86 g), MnCl_2_ × 4 H_2_O (1.81 g), ZnSO_4_ × 7 H_2_O (0.22 g), CuSO_4_ × 5 H_2_O (0.08 g), Na_2_MoO_4_ × 2 H_2_O (0.025 g), and CoCl_2_ × 6 H_2_O (0.025 g). One liter of the chelated iron stock solution (200×) contained FeSO_4_ × 7H_2_O (5.56 g) and Na_2_EDTA (7.45 g). A volume of 2.5 mL was added to the half-strength Hoagland’s solution. The pH was adjusted to 5.5 using KOH. Growth medium levels were checked daily and replenished regularly in each unit to maintain a stable water level. In addition, pH and EC (1.3 dSm^−1^) were monitored every 2–3 days and adjusted by replacing the medium with fresh solution. Plants were harvested on DAG 21. We used a modified Hoagland’s solution containing KH_2_PO4 instead of NH_4_H_2_PO_4_, as KH₂PO₄ offers greater pH stability in hydroponic systems. Moreover, the accumulation of ammonium can harm root development. Lettuce, spinach, and cai xin are known to be sensitive to elevated ammonium levels over time, particularly under higher temperatures conditions.

### Growth conditions during stress experiments

All seedlings were germinated on cotton, transferred to the Aspara unit for growth, and on DAG 5, the growth medium was replaced with various nutrient solutions or stress treatments were applied. Nutrient stress conditions were induced by modifying the growth medium (see **Tables 1**–**3**). For cai xin and lettuce, control growth conditions included a light intensity of 202.5 μmol·m^−2^·s^−1^, an R:B:W ratio of 4:1:1, and a 20-hour photoperiod at 25°C. For spinach, the control conditions consisted of a light intensity of 130 μmol·m^−2^·s^−1^, an R:B:W ratio of 4:1:1, and a 15-hour photoperiod at 22°C.

In the light intensity experiment, cai xin and lettuce were grown under light intensities of 67 μmol·m^−2^·s^−1^, 135 μmol·m^−2^·s^−1^, 202.5 μmol·m^−2^·s^−1^, and 268 μmol·m^−2^·s^−1^ with a 16-h photoperiod at 25°C. Spinach was grown under light intensities of 65 μmol·m^−2^·s^−1^, 130 μmol·m^−2^·s^−1^, 200 μmol·m^−2^·s^−1^, and 260 μmol·m^−2^·s^−1^ with a 15-hour photoperiod at 22°C. In the photoperiod experiment, cai xin and lettuce were grown at 8 h, 12 h, 20 h, and 24 h light at 25 °C, 200 μmol·m^−2^·s^−1^ and R:B:W 4:1:1. For spinach, photoperiods of 8 h, 13 h, 18 h, 24 h were tested at 22 °C, with a light intensity of 130 μmol·m^−2^·s^−1^ and an R:B:W ratio of 4:1:1. In the light quality experiments, R:B:W ratios of 4:1:1, 4:1:0, 3:1:1, and 3:1:0 were applied to all plant species under otherwise control conditions. For the temperature experiments, all plant species were grown at 20°C, 25°C, 30°C, and 35°C under otherwise control conditions.

In the modified N solution (**Table 1**), KNO_3_ and Ca(NO_3_)_2_ were replaced with KCl and CaCI_2_, respectively, to maintain equivalent concentrations of K and Ca as in the original formulation. In the modified P solution (**Table 2**), KH_2_PO4 was replaced by KCl, while in the modified K solution (**Table 3**), KH_2_PO4 and KNO_3_ were substituted with NaH_2_PO4 and NaNO, respectively_3_. Although careful efforts were made to ensure ion concentrations were equivalent across all complete and nutrient-deficient media formulations, some variance still exists. Therefore, while all treatments were normalized against their respective growth controls to minimize within-treatment variability, cross-treatment comparisons should be interpreted with caution due to potential residual differences.

**Table 1:** Composition of modified nitrogen (N) solutions at different concentrations (0–150%).

Modified N Solution	0% N (μM)	25% N (μM)	50% N (μM)	100% N (μM)	150% N (μM)
KNO_3_	0	750	1,500	3,000	4,500
KCl	3,000	2,250	1,500	0	0
Ca(NO_3_)_2_	0	500	1,000	2,000	3,000
CaCI_2_	2,000	1,500	1,000	0	0

The table shows the concentrations (in μM) of KNO_3_, KCl, Ca(NO_3_)_2_, and CaCl_2_ used to create solutions with 0%, 25%, 50%, 100%, and 150% nitrogen levels.

**Table 2:** Composition of modified phosphorus (P) solutions at different concentrations (0–150%).

Modified P solution	0% P (μM)	25% P (μM)	50% P (μM)	100% P (μM)	150% P (μM)
KH_2_PO4	0	125	250	500	750
KCl	500	375	250	0	0

The table displays the concentrations (in μM) of KH_2_PO_4_ and KCl for generating phosphorus solutions across five concentration levels.

**Table 3:** Composition of modified potassium (K) solutions at different concentrations (0–150%).

Modified K Solution	0% K (μM)	25% K (μM)	50% K (μM)	100% K (μM)	150% K (μM)
KH_2_PO4	0	125	250	500	750
NaH_2_PO4	500	375	250	0	0
KNO_3_	0	750	1,500	3,000	4,500
NaNO_3_	3,000	2,250	1,500	0	0

The table outlines the concentrations (in μM) of KH_2_PO_4_, NaH_2_PO_4_, KNO_3_, and NaNO_3_ used to prepare potassium solutions at varying concentrations.

NaOH was used to adjust the pH to 5.5.

### Sampling of plants and determination of fresh weight

On DAG 21, out of the five biological replicates per condition, the three most healthy biological replicates with similar growth characteristics were selected. Three to four mature leaves were cut, weighed (minimum total weight 0.5 g), immediately flash-frozen in liquid nitrogen, and stored at −80°C. After RNA sampling, the rest of the leaves were cut, and the fresh weight (FW) was measured to obtain the total fresh weight of each biological replicate.

### RNA isolation and sequencing

All leaf samples were ground with a mortar and pestle, and the frozen powder was aliquoted and stored at −80°C until use. RNA was isolated from stressed and control plants (three biological replicates each) using a Spectrum™ Plant Total RNA Kit (Sigma). Quality control of all extracted RNA (triplicates for each condition) was performed by Novogene (Singapore) using Nanodrop and agarose gel electrophoresis (for purity and integrity) before sample quantitation and further analyses of integrity (Agilent 2100 Bioanalyzer). The library type was a eukaryotic directional mRNA library. Library construction from total RNA, including eukaryotic mRNA enrichment by oligo(dT) beads, library size selection, and PCR enrichment, was performed using a Novogene using NEBNext^®^ Ultra™ II Directional RNA Library Prep Kit for Illumina^®^. The libraries were sequenced with NovaSeq-6000, paired-end sequencing at 150 base pairs and at sequencing depths of approximately 20 million reads per sample (6 Gb/sample).

### Gene expression and differential gene expression estimation

Transcript abundance from RNA-sequencing data was quantified using kallisto v0.46.1 (Bray et al., 2016). Pseudoalignment was performed against the CDSs of *Brassica rapa* (BRADv1.2 Cheng et al., 2011; Chen et al., 2022), *Lactuca sativa* (V8 Reyes-Chin-Wo et al., 2017), and *S. oleracea* (Spov3 Hulse-Kemp et al., 2021) all obtained from Phytozome (Goodstein et al., 2012). Gene expression output from kallisto includes count and TPM (transcripts per million). Pearson correlation coefficients (PCCs) was calculated based on TPM values to assess expression similarities across replicates and stress treatments. For each species, clustermaps were generated to visualize correlations, and sample similarities were further analyzed using Euclidean distances.

Differential gene expression was determined using DESeq2 v1.40.1 (Love et al., 2014) with count outputs from kallisto. For the various stress conditions, comparisons were made against their respective stress experiment controls. Genes were identified as differentially expressed at $\left|log_{2}FC\right|$ ≥ 1 and adjusted p-value ≤ 0.05 (corrected using the Benjamini–Hochberg procedure).

### Gene annotation and differentially expressed biological functions

The biological function annotations of the genes from all three species were assigned using Mercator4 v5.0 (Schwacke et al., 2019), where each annotation refers to a MapMan bin. Transcription factors (TFs) and their corresponding gene families for cai xin and lettuce were identified using iTAK v1.6 (Zheng et al., 2016), while those for spinach were identified using iTAK v1.7.

Survival function was used to identify significantly differentially expressed biological functions, with significance defined as a Benjamini–Hochberg (BH) adjusted p-value of less than 0.05. To infer similarities between biological functions (rows) and stress conditions (columns), Jaccard distances (JDs) were computed between them, respectively. These JDs were then used to perform hierarchical clustering of both biological functions and stress conditions.

### Pathway correlation with Normalized Differential Expression Index analysis

Differential expression of a biological function within each species was quantified using the Normalized Differential Expression Index (NDEI), which indicates the activation or repression of a given MapMan bin under each stress. NDEI is defined as the normalized difference between the number of up- and downregulated genes under each stress condition, relative to the MapMan bin size, as follows:


$$NDEI\left(\sigma ,m\right)\;=\;\frac{U\left(\sigma \right)\;-\;D\left(\sigma \right)}{B\left(m\right)}$$

where *U* and *D* represent the total number of up- and downregulated genes, respectively, under stress condition σ , and *B* is the total number of genes assigned to MapMan bin *m*. This index enables direct comparison of functional responses across different stresses and species by normalizing for pathway size. Positive NDEI values indicate predominant upregulation of a biological process, while negative values reflect dominant repression. As such, NDEI highlights the overall transcriptional shift of each biological process in a consistent and interpretable way.

The correlation between pairs of biological functions was assessed using Pearson correlation coefficients (PCCs), computed between all MapMan bins at the same level based on their respective NDEI values. A pair of biological functions was determined to be conserved when they were significantly differentially expressed in a similar manner (up- or downregulated) under the same surplus or deficient stress condition—where *surplus* referred to an increase in the stress parameter relative to the control, and *deficient* the inverse—in at least two species.

### Detection of orthogroups and calculation of significant similarities between stresses

Orthologues were inferred with OrthoFinder v2.5.5 (Emms and Kelly, 2015; Emms and Kelly, 2019), through which differentially expressed orthogroups were identified by mapping differentially expressed genes (DEGs) to their orthogroups. To assess the similarity of stress responses within and between species, we used the Jaccard Index (JI), a standard measure of set similarity. It quantifies the proportion of shared differentially expressed genes or orthogroups between conditions, offering a straightforward and interpretable way to compare transcriptional responses across multiple datasets. For comparison of stress conditions within a species, the JI was computed between all stress conditions using the DEGs datasets. For cross-species comparison, JI was computed between all stress conditions and species based on the differentially expressed orthogroups. The significance of the quantified similarities was evaluated through permutation analysis, where observed JIs were compared to permuted JIs. A thousand permutations were executed for each observed JI, and significance was assumed at BH p-adjusted <0.05. Conserved orthogroups were defined as those differentially expressed in all three species and in a similar manner (up- or downregulated) under the same surplus or deficient stress condition.

Based on the conserved orthogroups, the conserved DEGs in all three species were queried against *A. thaliana* (Araport11 Cheng et al., 2017; TAIR: The Arabidopsis Information Resource) using BLAST (Camacho et al., 2009), a local sequence similarity tool, to identify their best hits. A literature review was subsequently conducted on these best hits to determine their experimentally verified functions and assess their relevance to the stresses analyzed in this study.

### Construction of gene regulatory networks

We used GENIE3 (Huynh-Thu et al., 2010; Aibar et al., 2017), a machine learning algorithm based on random forests, to construct gene regulatory networks (GRNs) for individual species using the TPM expression values of DEGs. By predicting which transcription factors regulate which target genes, GENIE3 enables robust identification of regulatory interactions, especially when direct perturbation data are unavailable. TFs were specified as the regulators. For high-confidence networks, the top 5% of TF-target edges (ranked by non-zero weight) were used for downstream analyses. The relationships between TFs and their target genes were assessed using PCC analysis of gene expression data (TPM), where positive and negative correlations indicated TFs acting as activators and repressors, respectively.

Stress-specific GRNs were constructed using conserved TF-target edges. Similar to the identification of conserved orthogroups, TF-target edges were first mapped to their respective orthogroups for cross-species comparison. Conserved TF-target edges were defined as those differentially expressed in a similar manner (up- or downregulated) under the same surplus or deficient stress condition in more than one species. The significance of the conserved edges was evaluated through permutation analysis, comparing the observed conserved edges to permuted ones. The enrichment score was calculated as the ratio of observed conserved edges to the mean of permuted conserved edges. Significance was assumed when the p-value (corrected using the BH procedure) was <0.05 and the enrichment score >1. Henceforth, TFs from significantly conserved TF-target edges were referred to as “the stress-specific conserved TFs.”

### Identifying biological functions of stress-specific conserved TFs

The biological functions of TFs were predicted by identifying the MapMan bins of their target genes. The number of target genes assigned to each biological function was normalized by the corresponding MapMan bin size. For each species, the normalized values of all TFs linked to a specific gene family were summed and then divided by the total number of TFs associated with that gene family for the respective biological function. This process was repeated for each biological function across the three species. The regulatory strength of a gene family for a biological function across species was calculated as the average of these species-specific values, expressed as:


$$R\left(m,g\right)=\frac{1}{S}\sum_{i=1}^{S}\frac{\sum_{j=1}^{T_{i\;}}\frac{t\left(m,\;g\right)}{B\left(m\right)}}{T_{i}}\;,$$

where *t* is the number of target genes of conserved TF *j* of gene family *g* assigned to MapMan bin *m*, *T_i_* is the total number of conserved TFs of gene family *g* in species *i*, and *S* = 3 is the total number of species.

### Stress-specific conserved TF response with experimentally verified Arabidopsis genes

The stress-specific conserved TFs that regulated at least five biological functions were queried against *A. thaliana* using BLAST to identify their best hits. The stress conditions under which the conserved TFs (columns) were differentially expressed under were mapped against the experimentally verified functions of the best BLAST hits (rows) based on their Gene Ontology (GO) terms; a match indicated conservation of the TFs’ roles in *A. thaliana*. The *A. thaliana* best BLAST hits (rows) were grouped by function, with the miscellaneous category including GO terms such as “response to hydrogen peroxide,” “response to wounding,” “cellular response to hypoxia,” “response to water deprivation,” and other functions not represented as separate categories.

Permutation analysis was performed to evaluate the significance of functional conservation between the predicted roles of the stress-specific conserved TFs and the functions of their *A. thaliana* best BLAST hit functions. The observed matches were compared to permuted matches from 1,000 permutations of the stress conditions under which the conserved TFs were differentially expressed, while keeping the best hits and their experimentally verified functions constant. Significance was assumed when the p-value (corrected using the BH procedure) was <0.05.

### Establishment of StressPlantTools database

Using the CoNekT framework admin panel (Proost and Mutwil, 2018), we constructed the database from the generated gene expression data. We employed the Highest Reciprocal Rank metric to construct the coexpression networks (Mutwil et al., 2010). For each species, coexpression clusters were obtained using the Heuristic Cluster Chiseling Algorithm (HCCA). The database is hosted on an Apache server running Windows OS.

### Data availability

The raw sequencing data are available at ENA under accession number E-MTAB-14018.

## Results

### Growth responses of lettuce, cai xin, and spinach under abiotic stresses reveals differential requirements for optimal growth

The hydroponic crops lettuce, cai xin, and spinach are grown mainly for their shoot portions, thus, we aimed to understand how various abiotic and nutrient stresses affect their growth and functional phenotypes. To determine the effects of environmental conditions on these crops, plants were grown for 16 days under their respective experimental conditions (**Figure 2A**), and phenotypes and fresh weights (FWs) were recorded on DAG 21 (days after germination; **Figure 2B**; **Supplementary Table S1**). The control medium for all plants was half-strength Hoagland solution, a widely used standard growth medium characterized by high levels of N and K, making it suitable for plants with high nutrient demands.

**Figure 1:**
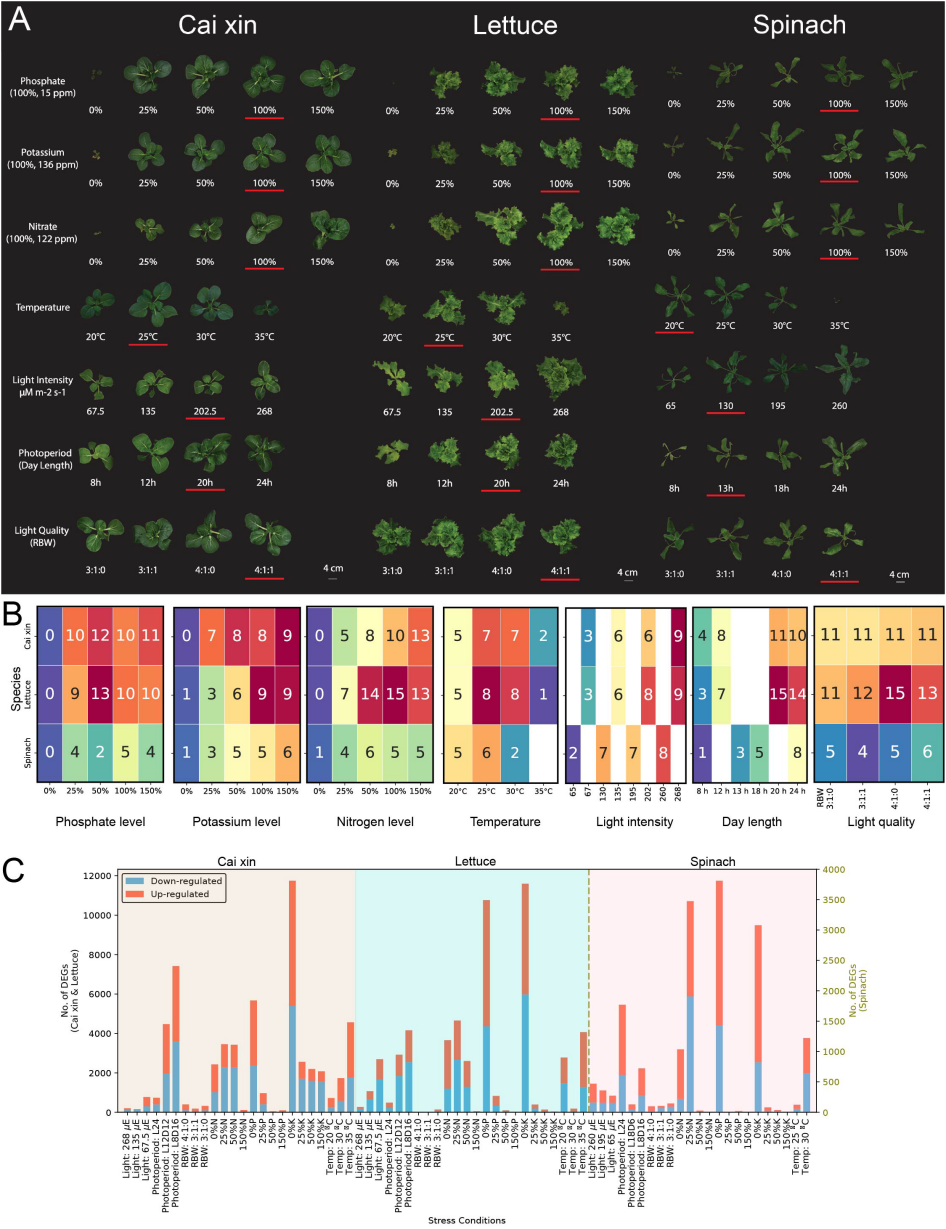
Abiotic stress experiments for hydroponically grown cai xin, lettuce, and spinach. **(A)** Phenotypes of cai xin, lettuce, and spinach on DAG 21 under phosphate, potassium, nitrate, temperature, light intensity, photoperiod, and light quality stress experiments. The control condition of each stress is underlined. **(B)** The mean fresh weight data (rounded to the nearest gram) from triplicates are annotated in the cells of the heatmaps for phosphate, potassium, nitrate, temperature, light intensity, photoperiod, and light quality stress experiments. **(C)** Differential gene expression analysis of cai xin, lettuce, and spinach. The red and blue bars represent upregulated and downregulated genes, respectively. The x-axis represents different stress conditions while the y-axes show the number of differentially expressed genes (DEGs) for a given stress (left y-axis for cai xin and lettuce; right y-axis for spinach).

**Figure 2:**
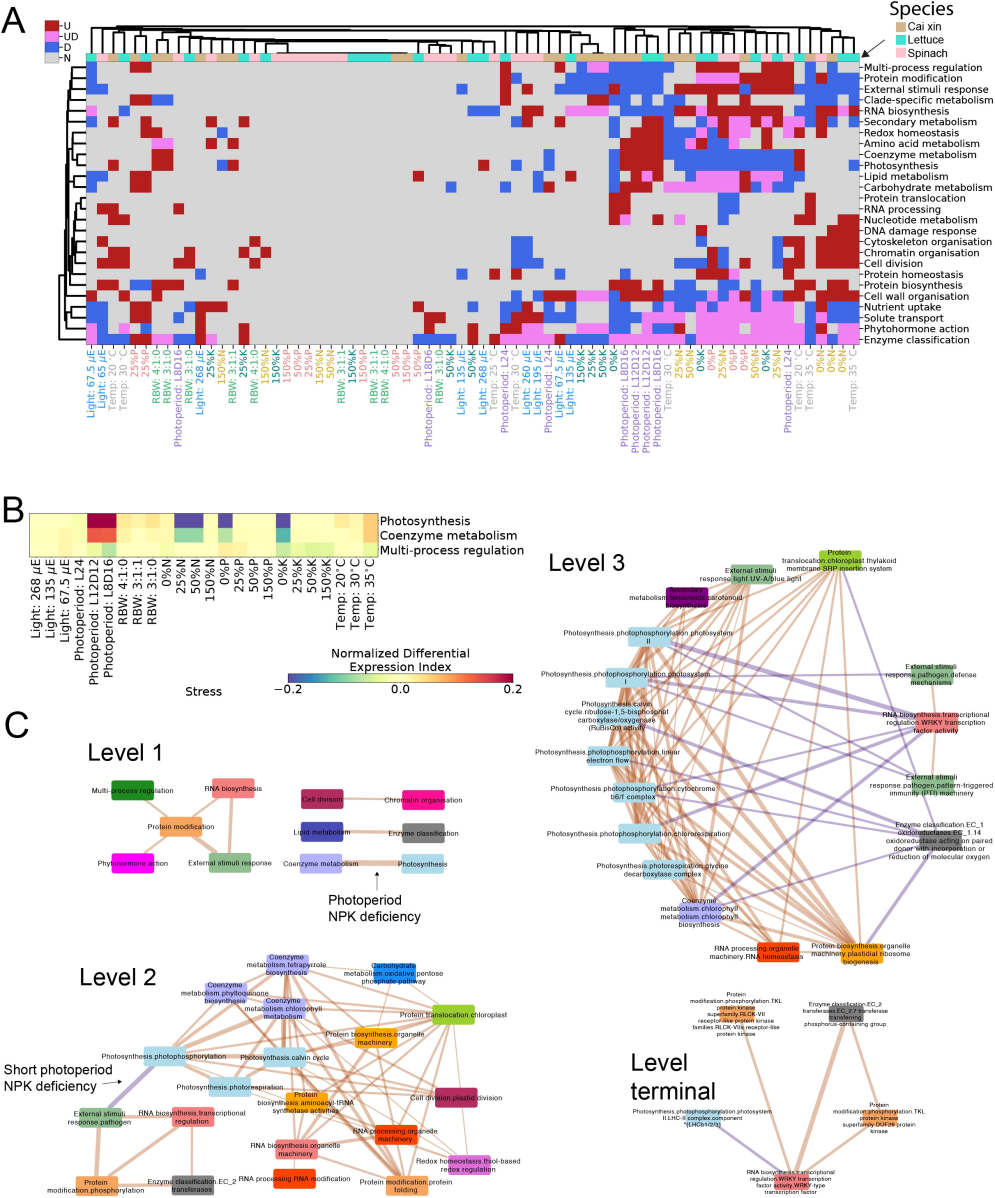
Expression profiles and co-expression analyses of stress responses. **(A)** Significantly (BH p-adj <0.05) upregulated (red), downregulated (blue), or both (pink) biological functions for the different stress conditions and species. The different stress conditions are shown in columns, while the biological functions are in rows. Only biological functions that were differentially expressed in three or more columns are shown. Similarities between the responses were highlighted by clustering the columns and biological functions across all three species, with the columns color-coded such that tan, turquoise, and pink represent cai xin, lettuce, and spinach, respectively. The labels of the stress conditions are also colored according to their respective stress experiments. **(B)** Normalized Differential Expression Index (NDEI) values of photosynthesis, coenzyme metabolism, and multi-process regulation of cai xin under various stress conditions, where red and blue represent positive and negative NDEI values, respectively. **(C)** Correlations between biological functions defined by various levels (levels 1, 2, 3, and terminal) of MapMan bins. The biological functions are depicted as nodes and color-coded according to level 1 bins. The thickness of the edges represents the number of stresses in which the relationship is conserved. Edge colors represent positively correlated functions in orange and negatively correlated functions in purple. For brevity, only correlations conserved across all three species and in at least five conditions are shown.

Complete removal of a macronutrient from the growth medium—0% N, 0% P, or 0% K—resulted in severely reduced growth of all three species (**Figures 2A, B**). In media with reduced P and K macronutrients (25%, 50%; **Figure 2B**), cai xin displayed FWs similar to the control (100%). In contrast, it was highly sensitivity to reduced N levels, with fresh weight reaching 13 g at 100% (**Figure 2B**). This indicates that cai xin depends on high N—but not P or K—for better growth. Lettuce grew well at lower P levels (25%, 50%) but showed decreased growth under low K (25%, 50%) and low N (25%). Spinach grew better with increasing K concentrations (**Figure 2B**) but showed variable fresh weights at different P and N levels. Increasing nutrient concentrations (N, P, and K) to 150% did not significantly enhance growth in any species. Overall, these results show that the three species have different requirements for optimal fresh weight.

Temperature played an important role in achieving maximum FW. Both cai xin and lettuce grew well at 25°C and 30°C but showed reduced growth at 20°C and 35°C (**Figures 2A, B**). Spinach grew best at 25°C but was more sensitive to heat at 30°C and 35°C. While cai xin and lettuce could still grow at 35°C, spinach plants died after a few days (**Figures 2A, B**).

Low light intensities (65 μmol·m^−2^·s^−1^ to 135 μmol·m^−2^·s^−1^) and shorter photoperiod (8 h to 13 h of light) significantly decreased growth of all three species compared to controls. In cai xin and lettuce, the highest light intensity (268 μmol·m^−2^·s^−1^) and longest photoperiod (24 h light) did not promote further growth (**Figure 2B**). In contrast, spinach showed maximum FW at the highest light intensity (260 μmol·m^−2^·s^−1^) or the longest photoperiod (24 h light). However, some leaves showed yellowing at the tips and curling (data not shown). Thus, a lower light intensity of 130 μmol·m^−2^·s^−1^ and a shorter photoperiod of 13 h light were chosen as the default for spinach in other experiments, consistent with previous reports (Zou et al., 2020). Variation in light quality by modifying ratios of red, blue, and white light did not cause significant phenotypic changes in any of the three species (**Figure 2B**).

### Nutrient deficiencies and photoperiod changes induce major gene expression shifts

To better understand how the three species respond to different growth conditions at the gene expression level, we performed RNA-sequencing. For each of species and stress conditions, gene expression data were generated in triplicates (**Supplementary Data 1**–**3**) for expression matrices of cai xin, lettuce, and spinach, respectively). A total of 276 RNA-seq samples (**Supplementary Table S2**) were obtained across 31 stress conditions (30 for spinach, as the plants died at 35°C) from all three species. Clustering of the samples revealed high similarity among nearly all replicates, and distinct clustering patterns for N, P, and K deficiencies in cai xin (**Supplementary Figure S1**), lettuce (**Supplementary Figure S2**), and spinach (**Supplementary Figure S3**), indicating a strong transcriptional response to these stresses.

To determine the genes that exhibit differential expression in various stress conditions, differentially expressed genes (DEGs) were identified using DESeq2 for all stress conditions and species (**Figure 2C**; **Supplementary Tables S3**–**S5**). Among the species analyzed, spinach demonstrated the fewest DEGs (<4,000 DEGs across all stress conditions), while the other two species exhibited a higher number (12,000 DEGs). Deficiencies in N, P, and K (0% to 50%) induced the highest number of DEGs in all species. Conversely, surplus levels of N, P, and K (150%) had minimal effect on gene expression, with the exception of 150% K in cai xin, which showed 2,080 DEGs.

While light quality resulted in a low number of DEGs for all species (<397, **Figure 2C**), changes in light intensity and photoperiod had pronounced effects on the gene expression. Lettuce showed a large number (2,690) of DEGs under low light (67.5 μmol·m^−2^·s^−1^). A shorter photoperiod induced a high number of DEGs in cai xin (7,421 DEGs, 8 h light) and lettuce (4,160 DEGs, 8 h light). Conversely, a long photoperiod (24 h light) resulted in few DEGs in cai xin (732) and lettuce (485), but many in spinach (1,770).

Temperature variations had a prominent effect on the gene expression of all three species, with high temperatures (35°C for cai xin and lettuce, and 30°C for spinach) resulting in an increase in DEGs. Evidently, lettuce was sensitive to temperatures lower than the control, with a substantial number of DEGs reported at 20°C. Overall, nutrient deficiencies elicited the strongest transcriptional responses across all species, with nitrogen deficiency consistently inducing the highest number of differentially expressed genes. This trend aligns with the observed phenotypic data (**Figures 2A, B**), where nutrient deficiencies led to the most pronounced growth reductions. In contrast, nutrient surplus conditions generally provoked minimal changes in gene expression, suggesting that plants can tolerate elevated nutrient levels more easily than shortages. The magnitude of the transcriptional response mirrored the growth impairments seen under stress, reinforcing the tight coupling between physiological performance and underlying gene regulation. Light-related stresses, particularly shortened photoperiods and low light intensities also produced moderate to strong DEG responses, which matched their detrimental effects on fresh weight. Together, these results highlight that nutrient and light availability are dominant factors shaping transcriptional stress responses in hydroponic crops, both at the phenotypic and molecular levels.

### Conserved biological pathway responses revealed by cross-species gene expression analysis

**Figure 3:**
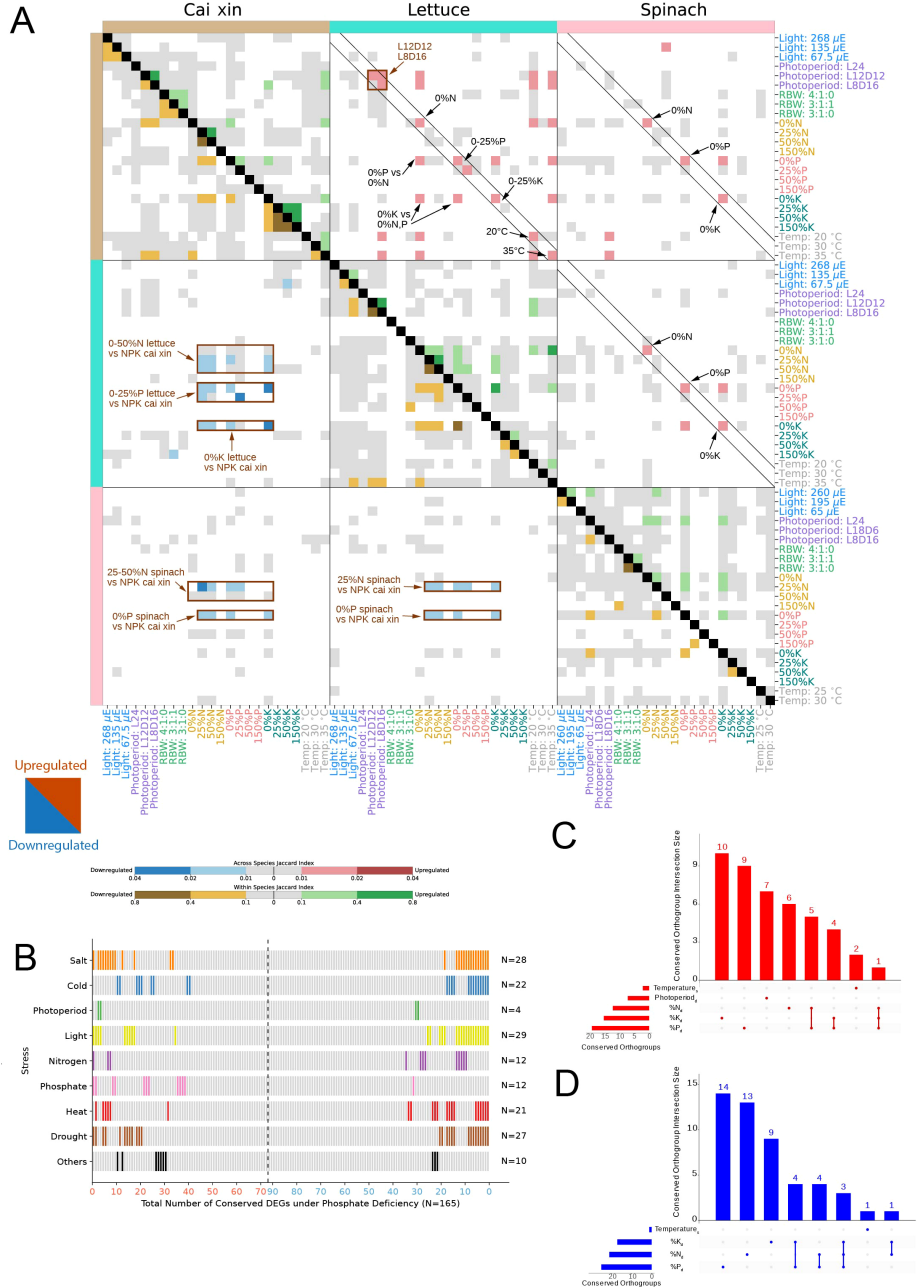
Conservation analysis of transcriptomic stress responses. **(A)** The heatmap shows the conservation of differentially upregulated (upper right triangle) and downregulated (lower left triangle) genes (and orthogroups) across the three species in the various stress conditions. For the across-species analysis, Jaccard index values between 0.01–0.02 (light red) and 0.02–0.04 (dark red) were computed between the upregulated orthogroups of two stress conditions. Similarly, Jaccard indices between 0.01–0.02 (light blue) and 0.02–0.04 (dark blue) indicate similarities between the downregulated orthogroups of two stress conditions. For the within-species analysis, Jaccard indices between 0.1–0.4 (light green) and 0.4–0.8 (dark green) were computed between the upregulated DEGs of two stress conditions. Similarly, Jaccard indices between 0.1–0.4 (light brown) and 0.4–0.8 (dark brown) indicate similarities between the downregulated DEGs of two stress conditions. Gray cells represent Jaccard indices of 0 to 0.01 and 0 to 0.1 for across- and within-species analyses, respectively. White cells indicate no significance (BH p-adj >0.05) for the Jaccard index computed between two stress conditions. **(B)** All 165 DEGs (*N*) conserved across three species under phosphate deficiency are shown in different columns (x-axis), where 73 are upregulated (red, left to right of the x-axis), and 92 are downregulated (blue, right to left of the x-axis). The experimentally verified functions of the conserved DEGs’ respective *Arabidopsis thaliana* best BLAST hits are shown in rows. The category ‘Others’ encompasses osmotic stress and light–dark cycle. Conserved DEGs with verified functions are represented as bars, color-coded according to the different stress categories, and unverified functions are depicted as gray bars. Upset plots for **(C)** upregulated and **(D)** downregulated conserved orthogroups across species. The x-axis indicates the different stress condition combinations, while the y-axis indicates the number of orthogroups in a given combination. Subscripts “d” and “s” represent deficient and surplus, respectively.

To gain insight into the biological functions affected by the stress conditions, we identified significantly differentially expressed MapMan bins. We observed high similarities among the N, P, and K deficiencies across all species, with coenzyme metabolism and photosynthesis being significantly downregulated (BH p-adj <0.5); solute transport, enzyme classification and phytohormone action being both significantly up- and downregulated; and protein modification and RNA biosynthesis significantly upregulated, indicating conserved responses (**Figure 3A**). In particular, under 0% N and 0% P stress conditions, certain biological functions were similarly regulated across all three species. Under 0% N, cytoskeleton organization, cell division, chromatin organization, and cell wall organization were upregulated, suggesting enhanced root developmental activity, likely promoting lateral root initiation and elongation to improve nutrient foraging (Forde and Lorenzo, 2001; Krouk et al., 2010). The downregulation of the response to external stimuli may reflect a growth-defense trade-off, whereby immune signaling is suppressed to conserve energy for growth (Huot et al., 2014). For 0% P, the downregulation of photosynthesis and coenzyme metabolism across species indicates reduced energy production and metabolic activity, consistent with the central role of P in ATP and NADPH synthesis (Plaxton and Tran, 2011). The upregulation of RNA biosynthesis suggests transcriptional reprogramming in response to P deprivation, enabling stress-specific gene expression (Misson et al., 2005).

The consistent enrichment of biological functions such as protein modification and RNA biosynthesis across N, P, and K deficiencies suggests that these pathways play a central role in orchestrating general stress adaptation mechanisms. Protein modification processes, such as phosphorylation and ubiquitination, are essential for fine-tuning signaling cascades and for activating or repressing key metabolic and defense pathways under stress (Stone, 2014). Similarly, RNA biosynthesis supports global transcriptomic reprogramming, prioritizing protective functions and suppressing growth-related genes (Baena-González and Sheen, 2008). These functions act as regulatory hubs—flexible yet conserved—enabling rapid and coordinated responses to environmental changes. Their evolutionary conservation likely stems from strong selective pressure to maintain core cellular infrastructure that supports plasticity in response to diverse abiotic stressors. Thus, their recurrence across species and stress types underscores their role as foundational elements of the plant stress response network.

### Correlation analysis unveils functional linkages between biological functions

Given that the stresses affect multiple biological functions that are likely functionally linked, we propose that the gene expression data provide an opportunity to better understand how these different processes are interconnected. To this end, we investigated the correlation of the Normalized Differential Expression Index (NDEI) values across the different stresses in the three species (**Supplementary Tables S6**–**S8**). NDEI values range from −1 (all genes assigned to the pathway are downregulated in a given stress) to 1 (all genes are upregulated), and biological pathways that are functionally linked should show high NDEI value correlations. For example, photosynthesis and coenzyme metabolism (comprising several cofactors important for photosynthesis) showed a strong NDEI correlation of PCC = 0.93 (p-adj = 2.65E−09) in cai xin (NDEI values of 0.28942 and 0.13217, respectively, under 8 h of light stress; **Figure 3B**; **Supplementary Table S6**), while multi-process regulation (comprising stress-responsive genes) tended to show a negative correlation (NDEI value of −0.07051 under 8 h of light stress; **Figure 3B**; **Supplementary Table S6**), which aligns with photosynthesis being negatively regulated by stress (Ferrari and Mutwil, 2020).

To better understand how the different biological functions are connected, we performed NDEI correlation analysis across the three species and investigated the conservation of the responses. From the level 1 MapMan bin network, we observed that all conserved correlations between biological functions were positive, and that protein modification was positively correlated with multiple biological functions across various stresses in the three species (**Figure 3C**; **Supplementary Table S9**).

The more fine-grained level 2 network showed that photosynthesis-related processes (light blue nodes), such as photophosphorylation, photorespiration, and the Calvin cycle, were significantly positively correlated with coenzyme metabolism processes—such as tetrapyrrole and chlorophyll metabolism (lilac nodes)—and with protein modification processes such as protein folding (orange). These relationships indicate that stress conditions significantly affect photosynthesis and chlorophyll production, a well-established phenomenon. Interestingly, a pathogen-specific response (green) was also induced under stress; it was strongly associated with protein phosphorylation (orange) and RNA biosynthesis (pink), but strongly negatively correlated with photophosphorylation in photosynthesis. While it is known that pathogen-specific responses—such as pathogen triggered immunity (PTI) and effector triggered immunity (ETI)—can lead to decreased photosynthetic efficiency, chlorosis, and cell death (Su et al., 2018; Nguyen et al., 2021), the regulatory mechanisms connecting pathogen response and photosynthesis remain poorly understood.

A more fine-grained level 3 network showed that various photosynthesis-related (light blue) and plastidal ribosomal machinery (orange) processes were highly interconnected and positively correlated with each other. Conversely, WRKY transcription factors (pink) were negatively correlated with various photosynthetic pathway processes, such as chlororespiration, photosystem I and photosystem II; this correlation was conserved across multiple stresses (thick edges). WRKY transcription factors also showed positive correlations with responses to biotic stresses (pathogen defense and PTI responses), likely reflecting their established roles as key regulators of downstream genes in both abiotic and biotic stresses (Khoso et al., 2022; Ma and Hu, 2024). The identity of the specific WRKY transcription factor(s) involved in this process may be inferred from their negative correlation with LHCB proteins (light blue), consistent with previous findings that WRKY40 represses LHCB expression (Liu et al., 2013). While some of these associations—such as those between stress and photosynthetic efficiency, or between growth and defense—are already known, our analysis allows us to pinpoint likely sites of interaction among these various major signaling modules.

### Identification of conserved abiotic stress responses across species

**Figure 4:**
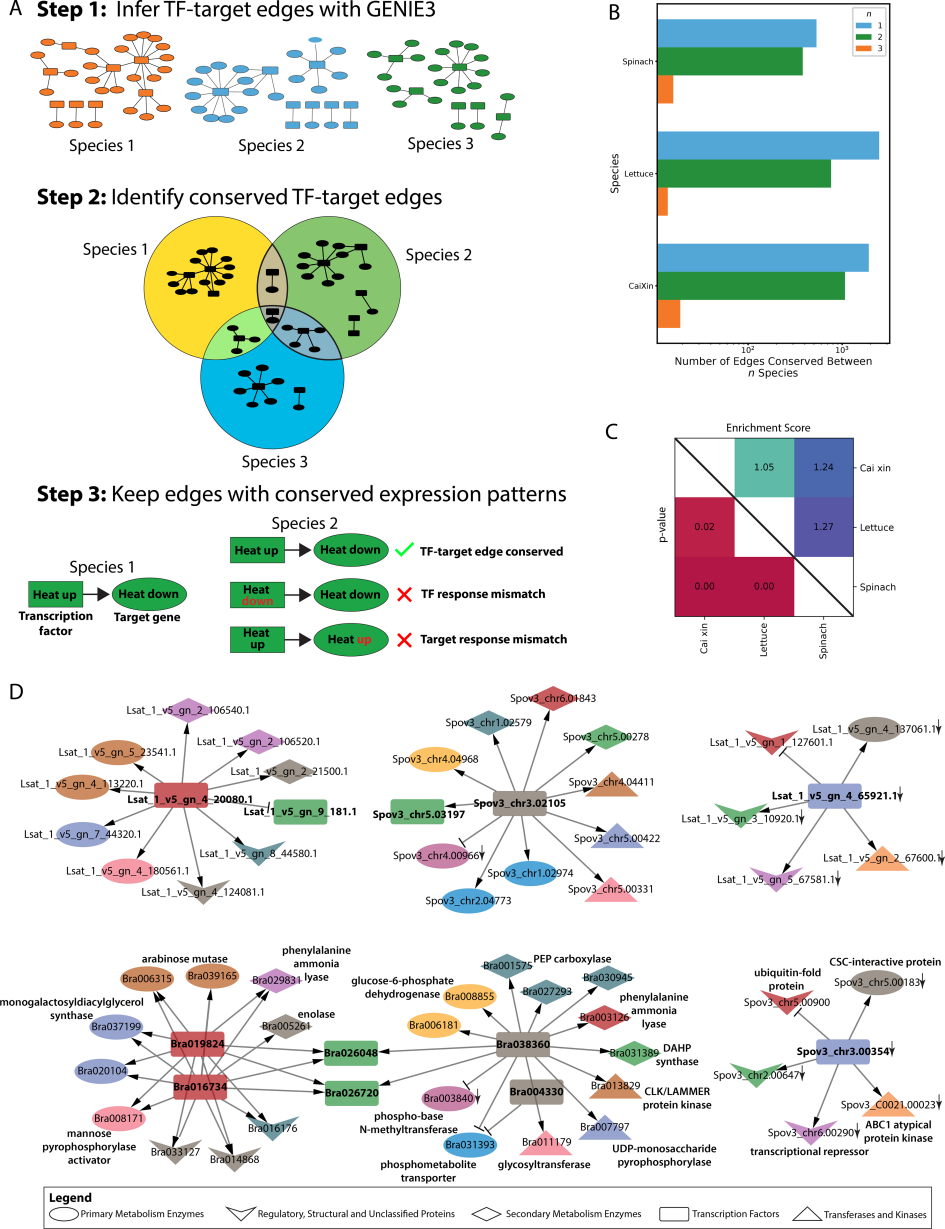
Construction and comparison of gene regulatory networks for cai xin, lettuce, and spinach. **(A)** Schematic workflow for identifying conserved TF-target edges. Step 1: GRNS were generated for each species using GENIE3, and the top 5% of TF-target edges (ranked by non-zero weight) were retained. Step 2: All TF-target edges were mapped to their respective OGs, and edges conserved across at least two species were identified. Step 3: Conserved TF-target edges under specific stress conditions were further refined by identifying edges that were consistently differentially expressed across species under the same stress. **(B)** Number of differentially expressed TF-target edges (top 5% by edge weight rank) in cai xin, lettuce, and spinach, categorized by the number of species in which they are conserved: blue bars (*n* = 1) for species-specific edges, green bars (*n* = 2) for edges conserved between two species, and orange bars (*n* = 1) for edges conserved across all three species. **(C)** Pairwise comparison of GRNs: adjusted p-values for edge conservation are shown in the lower right triangle, and enrichment scores (observed vs. expected overlap) are shown in the upper right triangle. **(D)** Conserved GARP TFs in phosphate deficiency GRNs across all three species, showing nodes of various shapes representing different functional protein types. Node colors correspond to different orthogroups. Delta and T-shaped arrows indicate activators and repressors, respectively. A down arrow (↓) indicates genes that are downregulated under phosphate deficiency.

Plants have evolved elaborate mechanisms to cope with stress, many of which are conserved to some extent across species (Ferrari and Mutwil, 2020; Wu et al., 2021; Leong et al., 2023). To better understand how stress responses are conserved across species, we investigated whether the three species exhibited significantly similar gene expression changes. Using permutation analysis we found that N, P, and K deficiency responses were significantly similar across species for downregulated genes (BH p-adj <0.05) (**Figure 4A**), brown rectangles, **Supplementary Tables S10**–**S13**). For instance, 0% N to 50% N stress conditions in lettuce elicited responses similar to N, P, and K deficiency conditions in cai xin (**Figure 4A**), lower left corner, blue cells). This shows that different stresses can elicit similar downregulation patterns.

For upregulated DEGs, identical stress conditions tend to upregulate similar sets of genes, particularly between lettuce and cai xin (upper right triangle, red squares found on the diagonal, **Figure 4A**). Furthermore, similar to the downregulated DEGs, N, P, and K deficiencies also upregulate comparable sets of genes across different nutrient deficiency conditions. For example, the 0% K stress condition in lettuce upregulated gene sets similar to those induced by 0% N and 0% P stress conditions in cai xin. In spinach, cross-species similarities in upregulated genes were also observed, although they were less frequent. Within each species, gene expression patterns were also observed across different stress conditions within the same stress experiment (**Figure 4A**), near main diagonal, yellow and green cells). Overall, we conclude that downregulated genes exhibit conserved but less condition-specific expression patterns compared to upregulated genes.

### Conserved upregulated genes are enriched for stress-related functions

To better understand the functions of the conserved upregulated genes, we performed a literature search of their best BLAST hits from *A. thaliana* (**Supplementary Table S14**). Several of the observed genes have already been reported to be involved in N, P, or K deprivation, such as a growth-regulating factor (Lantzouni et al., 2020), an R2R3 MYB transcription factor (Gaudinier et al., 2018), a calcium-dependent protein kinase (Qin et al., 2020; Liu et al., 2021; Adavi and Sathee, 2024) and other signaling components like the purple acid phosphatase *AtPAP12* (Wang et al., 2014). These conserved upregulated genes likely represent core regulators of abiotic stress responses, as they belong to TF families, kinases, and signaling enzymes that are known to act upstream in regulatory networks. Additionally, their consistent upregulation across three divergent species—and their previously validated functions in *A. thaliana*—suggest that they are part of a shared core stress signaling module. Thus, the genes in this list constitute valuable targets for studying how plants respond to abiotic stresses.

To investigate whether the identification of the conserved DEGs can enhance the inference of functionally relevant genes, we took a closer look at the experimental characterization of the Arabidopsis orthologs. A total of 165 DEGs were identified as conserved across all three species under phosphate deficiency, of which 73 were upregulated and 92 were downregulated (**Figure 4B**; **Supplementary Table S14**). The experimentally verified functions of these conserved DEGs, based on their best BLAST hits from *A. thaliana*, revealed that 76 DEGs have experimentally verified functions, and the majority were involved in light, salt and drought stress. Strikingly, nearly all genes that were verified to be involved in phosphate responses were upregulated (11 upregulated and one downregulated), further reinforcing the observation that upregulated—but not downregulated—genes tend to have stress-specific functions. Notably, eight upregulated and 18 downregulated DEGs were implicated in three or more stress responses, suggesting broader functionality of these genes and their role in fundamental regulatory networks. A substantial proportion of these genes were also implicated in other abiotic stress responses such as drought, light, and salt, further reinforcing their broad functionality and importance in integrating multiple environmental cues. Given their central regulatory roles and evolutionary conservation, these genes are attractive targets for translational applications. For instance, marker-assisted selection or genome editing to modulate their expression could lead to cultivars with enhanced resilience to combination stress without compromising yield—an outcome highly relevant for both field and controlled-environment agriculture.

### Functional analysis of conserved stress-responsive gene families

To identify gene families that might be important for stress responses, we identified orthogroups that exhibited conserved responses across all species under the deficient and/or surplus stress conditions (**Figures 4C, D**). In complement to **Figure 4A**’s observations, N, P, and K deficiency stress conditions encompassed the largest numbers of stress-specific, significantly conserved orthogroups, where 6, 9, and 10 upregulated, and 13, 14, and 9 downregulated orthogroups were identified, respectively. Notably, some conserved orthogroups show up- and/or downregulation in more than one surplus and/or deficient stress condition, as seen for N and P deficiencies (five upregulated and four downregulated orthogroups), K and P deficiencies (four upregulated and four downregulated orthogroups). and N, P, and K deficiencies (one upregulated and three downregulated orthogroups) (**Figures 4C, D**).

A total of 82 conserved orthogroups were identified under N, P, and K deficiencies, of which only nine had verified functions based on their best BLAST hits from Arabidopsis (**Supplementary Table S14**). Several of the conserved orthogroups were associated with phosphate acquisition and metabolism. Orthogroup OG0002122 (upregulated in P deficiency) encodes purple acid phosphatases 10 and 12 (PAP10 and PAP12), which are known for their roles in phosphate scavenging and recycling. These enzymes have been shown to enhance P deficiency tolerance by improving plant growth when overexpressed under P deficient conditions in *A. thaliana* and *B. napus* (Lu et al., 2008; Wang et al., 2011; Wang et al., 2014). Similarly, OG0002510 (upregulated in P deficiency) encodes phospholipase D zeta 1 (PLDζ1), which promotes root development under P deficiency by hydrolyzing phosphatidylcholine to release inorganic phosphate and support galactolipid synthesis (Li et al., 2006a; Li et al., 2006b). Lastly, OG0003037 (upregulated in P and K deficiencies) encodes glucose 6-phosphate/phosphate translocator 1 (GPT1), a gene critical for P stress adaptation in *B. napus* under low P conditions, and its Arabidopsis homolog, AtGPT1, also contributes to P efficiency under similar stress conditions (Yang et al., 2010).

Other conserved orthogroups function in stress signaling and redox regulation. OG0000492 (up- and downregulated in P and N deficiencies, respectively), encoding a catalase (CAT) enzyme, exhibited condition-dependent regulation—being repressed under N deprivation while induced by P starvation (Kandlbinder et al., 2004). OG0000699 (downregulated in N, P, and K deficiencies), corresponding to glutamine synthetase 2 (GS2), is a well-characterized nitrogen-responsive gene in barley, *Thellungiella halophila* and Arabidopsis (Kant et al., 2008; Schildhauer et al., 2008; Guiboileau et al., 2013). Similarly, OG0002621 (upregulated under P deficiency), which encodes glutamate decarboxylase 1 (GAD1), supports plant acclimation to P deficiency by upregulating the GABA shunt pathway and alleviating reduced 2-OGDH activity (Benidickson et al., 2023).

Conserved regulation was also observed among transcriptional regulators. OG0001470 (upregulated in P and N deficiencies), which encodes MYB62—a transcription factor known to suppress the expression of P starvation-induced genes—is specifically induced in leaves during P deprivation and regulates several aspects of the P stress response (Devaiah et al., 2009). OG0000576 (downregulated in P deficiencies), encoding indole-3-acetic acid inducible 14 (IAA14), is involved in lipid remodeling and P homeostasis under P deficiency in Arabidopsis (Narise et al., 2010).

Lastly, conserved regulation of ion transport was observed. OG000854 (downregulated in K deficiency) corresponds to K^+^ efflux antiporter 1 and 2 (KEA1 and KEA2), which are important for potassium homeostasis under K^+^ deficiency in *Arabidopsis*, with AtKEA1 being specifically expressed in both shoots and roots under low K^+^ stress (Zheng et al., 2013).

Taken together, this functional analysis of conserved stress-responsive gene families highlights how conserved gene families mediate nutrient sensing, signal transduction, transcriptional reprogramming, and ion transport during abiotic stress responses. Their consistent regulation across diverse species and nutrient conditions underscores their likely importance as central components of stress adaptation and suggests their potential utility in breeding programs aimed at improving nutrient stress resilience.

### Stress-responsive gene regulatory networks are conserved across species

Transcription factors are essential components of stress responses, and because they can control the expression of hundreds of target genes, they represent valuable targets for engineering stress adaptation. Because our dataset captures the stress responses of three species subjected to similar stresses, we developed a new approach that combines orthology and gene regulatory network analysis and identified conserved TF-target edges between two or more species (**Figure 1A**). From the GRNs of cai xin, 1071 TF-target edges were found to be conserved with the GRNs of either lettuce or spinach, and 19 TF-target edges were conserved with the GRNs of both lettuce and spinach (**Figure 1B**; **Supplementary Tables S15**–**S17**). Permutation analysis revealed that the TF-target edges were significantly conserved (BH p-adj <0.05) between two or more species, with especially strong conservation between cai xin and spinach, and between lettuce and spinach, where enrichment scores of 1.24 (i.e., 24% more edges than expected by random) and 1.27 were computed, respectively (**Figure 1C**).

Surplus- and deficiency-specific GRNs were built with significantly conserved TF-target edges for each species (**Supplementary Data 4**; **Supplementary Tables S15**-**S17**). In the conserved phosphate deficiency GRNs in cai xin, *Bra023066* (GRF) was observed to regulate 14 genes and two TFs—*Bra025775* (B3) and *Bra039733* (LOB) (**Supplementary Figure S4**). Additionally, *Bra023066* was regulated by four other TFs—*Bra000638* (HB), *Bra027050* (HB), *Bra017852* (AP2/ERF), and *Bra011782* (AP2/ERF) (**Supplementary Figure S4**). This reveals the complexity of the gene regulatory system, where multiple TFs are interconnected to coordinate responses to stressors. An independent network, consisting solely of GARP TFs—*Bra016734*, *Bra019824*, *Bra026048*, *Bra026720*, and *Bra038360*—was also observed under phosphate deficiency. Despite lacking interconnectivity with other TFs, they regulate a substantial number of genes, supporting previous findings that GARP TFs play an important role in phosphate signaling pathways (Safi et al., 2017; Yang et al., 2025).

A deeper investigation of the conserved GARP TFs, under phosphate deficiency GRNs across all three species revealed that, in cai xin, the GARP TF network formed a complete structure composed of two primary sub-networks (**Figure 1D**). These two sub-networks were primarily anchored by TFs from OG0002025 (*Bra016734* and *Bra019824*, depicted in red) and OG0006322 (*Bra004330* and *Bra038360*, shown in gray), which would otherwise function independently were it not for their connectivity to OG0001470 (*Bra026048* and *Bra026720*, colored green). The conservation of these two primary sub-networks—OG0002025 and OG0006322—was traced to lettuce and spinach, respectively. The complete network predominantly regulates enzymes involved in primary metabolism (e.g., arabinose mutase, monogalactosyldiacylglycerol synthase), secondary metabolism enzymes (e.g., enolase, DAHP synthase), as well as transferases and kinases (e.g., CLK/LAMMER kinase, glycosyltransferase). A conserved negative GARP network was also observed between lettuce and spinach only, in which the TFs activate a transcriptional repressor, an ABC1-atypical protein-kinase, and a CSC-interaction protein, while repressing a ubiquitin-fold protein. The partial overlap of these sub-networks highlights a unique yet conserved regulatory mechanism shared among the three species in response to phosphate deficiency.

### Regulatory strength identifies key transcription factors and associated biological functions under specific stress condition

**Figure 5:**
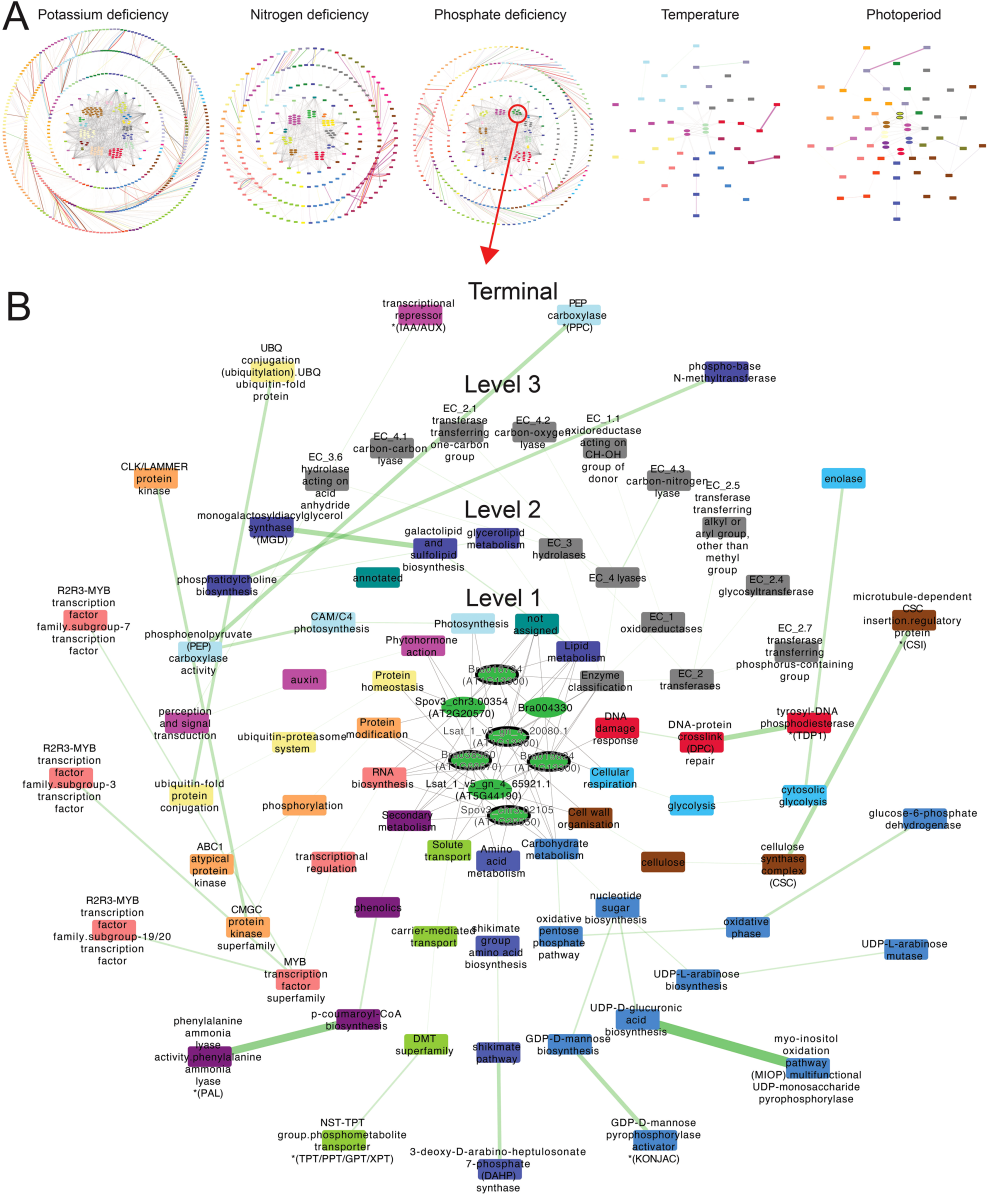
Transcription factor-biological process regulatory networks. **(A)** Networks of biological functions (MapMan bins) regulated by conserved TFs specific to potassium deficiency, nitrogen deficiency, phosphate deficiency, surplus temperature, and photoperiod deficiency-specific conserved TFs. **(B)** Regulatory network of conserved GARP TFs under phosphate deficiency, showing the biological functions governed by this TF family. The network is organized with conserved TFs from all three species positioned at the center of concentric circles. Each concentric layer represents a successive MapMan bin level, arranged radially from level 1 at the center to the terminal level on the outermost ring. Elliptical nodes represent TFs, while rectangular nodes represent biological functions, respectively. Gene families are distinguished by color, while biological functions are color-coded according to their corresponding level 1 MapMan bin. Each TF is connected to the level 1 MapMan bins it regulates. For deeper levels (levels 2, 3, and terminal), connections are established between adjacent MapMan bins levels (e.g., level 2 to level 1). An edge between two MapMan bins represents the regulation of a downstream function by a gene family, with edge colors indicating the corresponding gene family. The regulatory strength of a gene family for a given biological function is indicated by the weight of the edge, with thicker edges denoting stronger regulation.

To predict the biological functions of the conserved TF gene families, we analyzed the MapMan bins associated with their target genes. The resulting transcription factor–MapMan bin network illustrates which biological processes are likely regulated by TFs across multiple species under specific stress conditions. Potassium, phosphate and nitrogen deficiencies exhibited extensive conservation across two or more species, with a total of 150, 100, and 67 conserved TFs, respectively (**Figure 5A**; **Supplementary Table S18**). On the contrary, surplus temperature and shorter photoperiod exhibited lower conservation, with 14 and five conserved TFs, respectively (**Figure 5A**; **Supplementary Table S18**).

Among the conserved TF families, Growth-Regulating Factors (GRFs) were predominant under nitrogen deficiency, photoperiod deficiency, and surplus temperature, primarily regulating cell division, nucleotide metabolism, and DNA damage responses (**Supplementary Table S19**). These functions align with GRFs’ established roles in coordinating growth and developmental plasticity under fluctuating environmental conditions (Tu et al., 2024). Their strong regulatory activity under photoperiod stress, in particular, suggests that GRFs may serve as key levers for tuning plant architecture and biomass accumulation in low-light indoor farming systems.

In contrast, the WRKY and AP2/ERF TF families were predominant under potassium and phosphate deficiency. Under potassium deficiency, these TFs regulated amino acid metabolism, RNA biosynthesis, polyamine metabolism, and multi-process regulatory pathways. Under phosphate deficiency, they were associated with the regulation of coenzyme metabolism, protein modification, photosynthesis, redox homeostasis, and both RNA and protein biosynthesis. These findings are consistent with the well-documented roles of WRKYs and AP2/ERFs in nutrient stress signaling and broad transcriptome reprogramming (Jiang et al., 2017; Ma et al., 2024).

To examine more deeply the biological functions of GARP TFs under phosphate deficiency, we determined their regulatory strength—ranging from 0 to 1, where 1 indicates the target genes of a gene family from all species consistently regulate a biological function under specific stress—across various biological processes (**Figure 5B**). We observed high regulatory strength for phenylalanine ammonia lyase, UDP-monosaccharide pyrophosphorylase, DNA phosphodiesterase, and the R2R3-MYB TF family. The roles of the GARP TFs that regulate at least five biological functions were further validated against the experimentally confirmed functions of their best BLAST hit orthologs in *A. thaliana*, revealing that four of the seven TFs were responsive in phosphate starvation (Liu et al., 2009; Nagarajan et al., 2016; Ueda et al., 2020). Thus, our integrated analysis pinpoints transcription factors that govern specific stress-responsive pathways, highlighting genetic targets for improving crop resilience.

### Gene regulatory networks are conserved despite divergent biological functions across species

To verify whether the functions of the identified transcription factors are conserved in *A. thaliana*, we examined the experimentally validated functions of their best BLAST hit orthologs in *A. thaliana* (**Supplementary Figure S5A**; **Supplementary Table S20**). Only nine out of 139 (~6.5%) TFs exhibited conserved responses with their *A. thaliana* orthologs, the majority of which were associated with phosphate deficiency responses. A nitrogen-responsive hit was identified for *gn_4_20080.1*, which was downregulated under N deficiency and upregulated under P deficiency. Its best BLAST hit, *AT1G13300*, has also been shown to respond to nitrogen and phosphate starvation based on Gene Ontology annotations (Liu et al., 2009; Safi et al., 2021). *Bra016734* and *Bra019824* also share *AT1G13300* as their best BLAST hit, but only exhibit a conserved response under P deficiency. Notably, *Bra006085* and *gn_6_20120.1*, are differentially expressed under both N and photoperiod deficiency, yet their best hit, *AT5G11060*, has only been reported to be regulated by light (Serikawa et al., 1996). The mismatches between the conserved TFs and their *A. thaliana* orthologs suggest that the orthologs might have diverged in function or have not yet been studied under these specific stress conditions in *Arabidopsis*.

To assess whether the functions of the TFs are significantly conserved relative to *A. thaliana*, a permutation analysis was conducted. The results indicated significant conservation (BH p-adj = 0.001, **Supplementary Figure S5B**), with the observed proportion of functional matches exceeding that of the permuted matches (enrichment score = 1.67). However, although there is overall conservation of biological functions between the three crops and *Arabidopsis*, the conservation is primarily driven by shared responses to P and light.

### Identification of stress-related genes with StressCoNekT database

**Figure 6:**
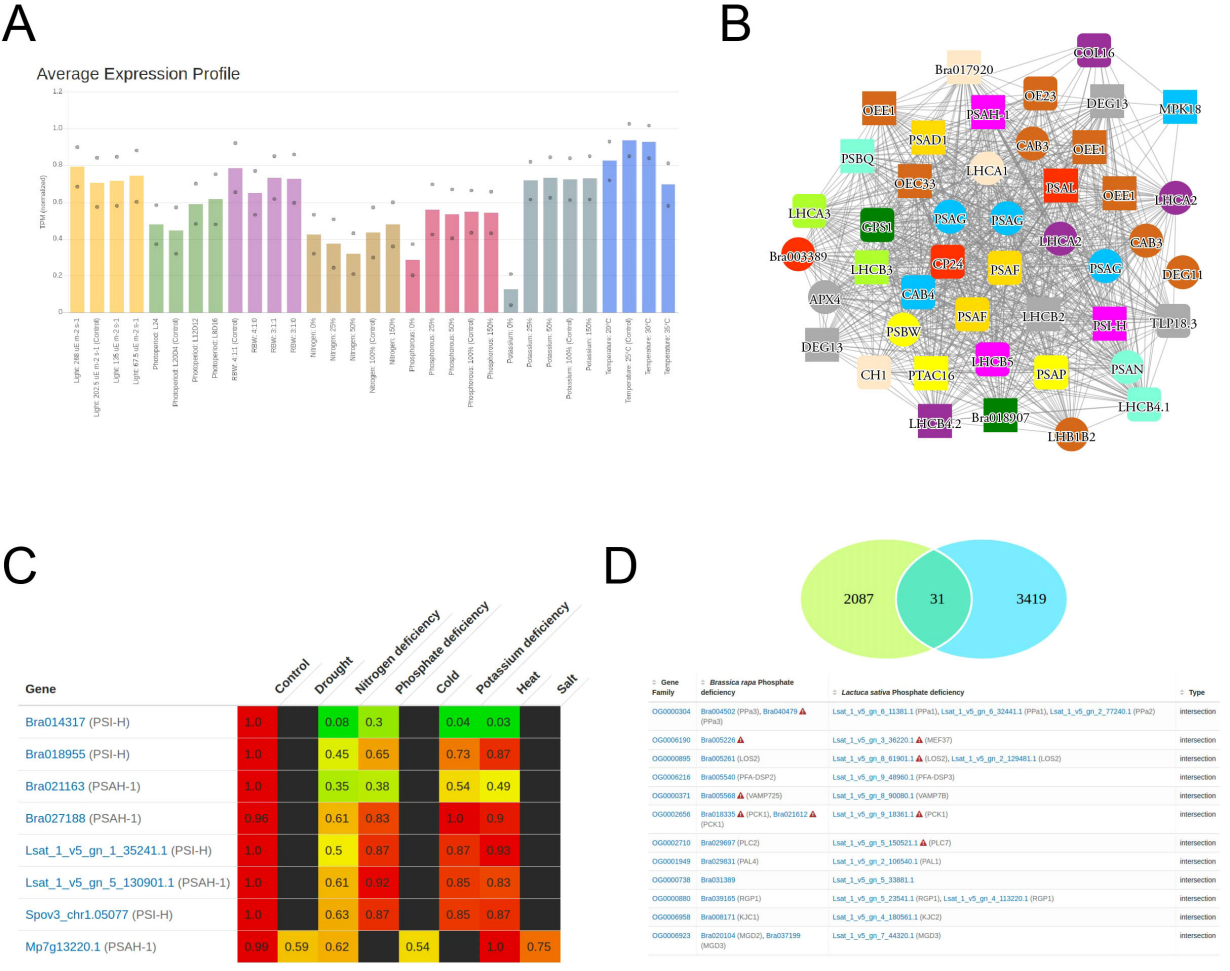
Example usage of StressCoNekT database. **(A)** Average expression profiles in the photosynthetic cluster. Colors indicate different stress experiments (x-axis), while average expression is shown on the y-axis. **(B)** Co-expression network of genes in the photosynthetic cluster. Nodes represent genes, edges connect co-expressed genes, and colored shapes represent different orthogroups. **(C)** Expression values of PSI-H genes in cai xin, lettuce, spinach, and *Marchantia polymorpha*. Genes are shown in rows, and stresses and controls in columns. **(D)** Venn diagram showing genes with conserved upregulation under phosphate deficiency in cai xin and lettuce.

The gene expression data for all stress experiments on the three species are made available on the StressCoNekT database (https://stress.plant.tools/), along with stress data for *Marchantia polymorpha* (Tan et al., 2023). The database serves as a platform for visualizing expression profiles, co-expression networks, and various comparative analyses. To demonstrate the utility of the database, we analyzed a co-expression cluster containing photosynthesis-relayed genes. The average expression profile (**Figure 6A**) revealed that photosynthetic gene expression was lower under N, P, and K deficiencies, consistent with what was observed in **Figure 3**). Meaningful co-expression networks can also be visualized; for example, the cluster included many known genes important for photosynthesis (**Figure 6B**). A comparative heatmap allows visualization of multiple gene expressions across stress conditions and species for comparison. For example, the photosystem I subunit H protein showed downregulation across all stresses, particularly during nitrogen deficiency (**Figure 6C**). Genes that show conserved responses can be identified using the ‘Compare specificities’ tool. In cai xin and lettuce under phosphate deficiency, 31 orthogroups were found to show conserved responses (**Figure 6D**). By cross-referencing the conserved orthogroup gene families with *A. thaliana*, six orthogroups (~20%) had been experimentally verified to have distinct roles under phosphate deficiency (**Table 4**). While the functions of the other genes on the list have not yet been reported as important for phosphate deficiency, their conservation strongly suggests a role in responding to this stress. In conclusion, the stress.plant.tools database enables the exploration of stress-specific expression profiles and will be an invaluable platform for stress-related studies.

**Table 4:** Phosphate starvation-specific orthogroups for *Brassica rapa* and *Lactuca sativa*.

Orthogroup	*Brassica rapa* gene ID	PubMed ID	*Lactuca sativa* gene ID	PubMed ID
OG0000304	Bra004502 (PPa3), Bra040479 (PPa3)		gn_6_11381.1 (PPa1), gn_6_32441.1 (PPa1), gn_2_77240.1 (PPa2)	
OG0006190	Bra005226		gn_3_36220.1 (MEF37)	
OG0000895	Bra005261 (LOS2)		gn_8_61901.1 (LOS2), gn_2_129481.1 (LOS2)	
OG0006216	Bra005540 (PFA-DSP2)		gn_9_48960.1 (PFA-DSP3)	
OG0000371	Bra005568 (VAMP725)		gn_8_90080.1 (VAMP7B)	
OG0002656	Bra018335 (PCK1), Bra021612 (PCK1)		gn_9_18361.1 (PCK1)	
OG0002710	Bra029697 (PLC2)		gn_5_150521.1 (PLC7)	
OG0001949	Bra029831 (PAL4)		gn_2_106540.1 (PAL1)	
OG0000738	Bra031389		gn_5_33881.1	
OG0000880	Bra039165 (RGP1)		gn_5_23541.1 (RGP1), gn_4_113220.1 (RGP1)	
OG0006958	Bra008171 (KJC1)		gn_4_180561.1 (KJC2)	
OG0006923	Bra020104 (MGD2), Bra037199 (MGD3)	31201686, 18808455, 16762032, 11553816, 21506606, 17419847, 14730084	gn_7_44320.1 (MGD3)	21506606, 18808455, 17419847, 16762032, 14730084, 11553816
OG0004400	Bra020624 (MYB78), Bra010021 (MYB78), Bra010022 (MYB78)		gn_6_70301.1 (BOS1)	
OG0002993	Bra033127, Bra014868		gn_6_113580.1, gn_4_124081.1	
OG0004295	Bra038357 (PHO1;H1), Bra004017 (PHO1;H1), Bra004334 (PHO1;H1)	17461783	gn_4_20480.1 (PHO1;H1)	17461783
OG0001200	Bra003102, Bra024235		gn_5_89840.1 (UUAT3)	
OG0000551	Bra018841 (STP9)		gn_6_7321.1 (STP1)	
OG0002152	Bra019824 (HRS1), Bra016734 (HRS1)	19341407, 29636481, 31811679	gn_4_20080.1 (HRS1)	19341407, 29636481, 31811679
OG0000634	Bra025150 (ALA10)		gn_8_37961.1 (ALA9), gn_4_17140.1 (ALA9)	
OG0002955	Bra026068 (GAPCP-2)		gn_7_110780.1 (GAPCP-1)	
OG0001609	Bra003443 (LMI2)		gn_7_5000.1 (LMI2), gn_3_120520.1 (MYB14), gn_8_50241.1 (MYB93)	
OG0000928	Bra012067 (GMD1)		gn_4_152760.1 (MUR1)	
OG0013618	Bra016176		gn_8_44580.1	
OG0000282	Bra007651		gn_2_87800.1	
OG0002253	Bra029482 (MEE51)		gn_0_27020.1	
OG0000963	Bra032846 (RSL4)	38267225, 1913310, 29651114, 30154812, 27427911	gn_7_20720.1 (RSL4), gn_5_114261.1 (RSL2)	38267225, 1913310, 29651114, 30154812, 27427911
OG0002936	Bra036222		gn_2_90021.1	
OG0003131	Bra037176 (PRX72)		gn_3_74860.1 (PRX72), gn_3_74880.1 (PRX72), gn_4_151960.1 (PRX72)	
OG0002255	Bra034307 (PAP12)	30341950, 24528675, 25270985, 20545876, 18716755, 12172020	gn_1_16540.1 (PAP12), gn_1_16580.1 (PUP3), gn_1_16600.1 (PAP12)	30341950, 24528675, 25270985, 20545876, 18716755, 12172020
OG0002810	Bra040330 (MAX3)	30466598	gn_3_130781.1 (MAX3)	30466598
OG0003095	Bra030749 (HAP5C)		gn_2_64201.1 (NF-YC3)	

For brevity, lettuce gene IDs have been shortened.

## Discussion and conclusion

Hydroponic cultivation is increasingly favored globally for its efficient resource management and the production of high-quality food. Traditional soil-based agriculture faces numerous obstacles, including urbanization, natural disasters, climate change, and the detrimental effects of excessive chemical and pesticide use, which diminish soil fertility (Sharma et al., 2019). Our studies show that, although plant growth often does not surpass control conditions, it is possible to reduce the levels of certain nutrients, such as PKN, to 50% of their recommended amounts without significantly impacting growth (**Figures 2A, B**). Beyond its resource efficiency, hydroponics serves as an invaluable research tool, enabling swift experimentation with various growth parameters. Our research demonstrated this capability by testing 24 growth conditions across three species simultaneously in a hydroponics-mimicking setup. Studying stress in a ‘production environment’ is critically important, given the low success rate in translating growth-promoting genes from models like *A. thaliana* to crops; of 1,671 genes tested in maize, only 22 (1.3%) yielded promising leads for further development (Simmons et al., 2021; Inzé and Nelissen, 2022). Unlike field experiments where environmental variables remain uncontrolled, hydroponics offers the flexibility to alter these parameters on the target crop in a parallelized manner, thereby enhancing the throughput, reproducibility, and reliability of research findings.

In recent years, nutrient deficiencies have emerged as significant threats to crop growth, production, food safety, and quality (Neset and Cordell, 2012; Shahzad et al., 2014). Prior research predominantly explored the mechanisms and signaling pathways that model plants, such as Arabidopsis and rice, employ to maintain homeostasis during individual nutrient shortages (Fan et al., 2021). These studies have enriched our understanding of the genes crucial for mineral nutrient balance under such deficiencies. Through molecular biology, genetics, and omics techniques, key regulators of nitrogen (N), phosphorus (P), zinc (Zn), and iron (Fe) absorption and equilibrium in *A. thaliana* and rice have been pinpointed during mineral scarcities (Kobayashi and Nishizawa, 2012; Park et al., 2014; Bouain et al., 2018; Yang et al., 2018). However, gene expression analyses, despite their value, often identify thousands of differentially expressed genes (**Figure 2C**), posing challenges in distinguishing genes important for stress survival from those that are merely secondary stress responses. This issue can be mitigated by comparative research, prioritizing genes with consistent expression patterns across species (Julca et al., 2023). For instance, conserved co-expression modules likely denote groups of truly functionally interconnected genes (Movahedi et al., 2011; Mutwil et al., 2011; Hansen et al., 2014). Our findings corroborate this approach, showing a high enrichment of genes essential for survival under phosphate deprivation survival in both cai xin and lettuce (**Table 4**; **Figure 6D**). Furthermore, we observed a unified response mechanism to NPK depletion across species (**Figure 4**), hinting at the potential for engineering resilience to these deficiencies by modifying the activity of the shortlisted genes.

The observation that downregulated genes were more broadly conserved yet functionally non-specific suggests a generalized stress response strategy, rather than one tailored to particular biological processes. This pattern may reflect a form of global gene expression suppression, a phenomenon also reported in basal algae such as *Cyanophora paradoxa* (Ferrari and Mutwil, 2020). In that study, genes downregulated across multiple abiotic stresses were associated with core biosynthetic processes and cellular functions, mirroring what has been observed in angiosperms. This supports the idea that repression of energetically demanding processes such as translation, transcription, and cell growth is an evolutionarily conserved feature of abiotic stress adaptation (Ferrari and Mutwil, 2020). In addition to energy conservation, the broad conservation of downregulated genes may also result from shared upstream signaling cascades that non-specifically suppress growth-related pathways in favor of defense and stress-mitigation programs (Baena-González et al., 2007; Nakashima et al., 2014). For example, stress-responsive pathways such as the MAPK cascade or ABA signaling are known to globally suppress transcription and translation machinery during the early phases of stress response (Kovtun et al., 1998; Aerts et al., 2024). Thus, the nonspecific but widespread downregulation of conserved genes across species likely reflects an ancient, coordinated mechanism for rebalancing cellular priorities under stress, rather than discrete functional modules being independently downregulated (Baena-González et al., 2007; Nakashima et al., 2014). The current analysis is based on bulk RNA-seq of whole leaf tissue, which may limit our understanding of how stress responses are coordinated across different tissues—and even within specific cell types. Future experiments investigating stress responses at the single-cell level using single-cell or spatial transcriptomics may help resolve how stress adaptation is coordinated across different cell types.

Correlation analyses of Normalized Differential Expression Index (NDEI) values further elucidate functional linkages between biological pathways. Positive correlations between photosynthesis and coenzyme metabolism, as well as protein modification and RNA biosynthesis, indicate coordinated regulation of these processes under stress conditions (**Figures 3B, C**). Our analysis revealed negative correlations between pathogen-specific responses and photosynthesis, suggesting an interaction between defense mechanisms and energy production, which aligns with the concept of growth-defense trade-offs (GDT) in plants. GDT is commonly observed in experiments on biotic stress and herbivory, in which defense signaling pathways are upregulated in response, resulting in the downregulation of growth-related pathways (Huot et al., 2014). MAP kinases, as first responders to pathogen-related effector molecules and other damage-associated molecular patterns (DAMPs) interact with WRKY transcription factors to suppress gibberellic acid signaling gene expression, stymying growth (Züst and Agrawal, 2017). RLKs and RLCKs have also been implicated in GDT, as many of these proteins serve as receptors for pathogenic molecules (Figueroa-Macías et al., 2021). While some associations—such as the relationship between stress and photosynthetic efficiency—are known, our analysis pinpoints likely sites of interaction among these major signaling modules. Here, we highlight the relationship between WRKY transcription factors and their connection with the photosynthetic component LHCB, without any *a priori* assumptions. However, we note that the observed pathogen-specific responses may, in part, be driven by WRKY-dominated patterns, suggesting that some findings could be biased due to the strong regulatory footprint of this TF family. The analysis effectively identifies relationships and components, offering meaningful perspectives on the intricate underlying mechanisms connecting various biological functions.

Stress adaptation mechanisms are orchestrated at the transcriptional level by transcription factors (TFs), leading to the accumulation of stress-responsive cellular factors (Ramanjulu and Bartels, 2002; Manna et al., 2021), thereby highlighting TFs as pivotal targets for genetic engineering. Leveraging the power of comparative transcriptomics and the parallel nature of our stress experiments, we have devised a novel pipeline that merges traditional regression methods with comparative genomics to construct conserved gene regulatory networks (**Figure 5A**). Our findings demonstrate significant conservation of these networks across species, delineating the biological pathways influenced by these TFs (**Figure 5**), yet they reveal limited conservation of functions attributed to Arabidopsis orthologs—except in phosphate and nitrogen deprivation (**Supplementary Figure S5**). This partial conservation may be attributed to the species-specific divergence of gene functions, a phenomenon also noted in animal studies (Berthelot et al., 2018). Additionally, morphological distinctions could influence the impact of certain genes. For instance, the *SAMBA* gene—a negative regulator of cell cycle progression—boosts leaf growth in Arabidopsis through enhanced cell division upon inactivation (Eloy et al., 2012; however, in maize, its mutation leads to reduced growth, possibly due to excessive cell division during later development stages (Gong et al., 2022a). This indicates that some growth-regulatory networks are exclusive to eudicots and absent in monocots—for example, the PEAPOD-KIX-TOPLESS repressor complex (Schneider et al., 2021), which limits growth in various eudicot organs (Naito et al., 2017; Cookson et al., 2022), but is absent in grasses (Schneider et al., 2021). Furthermore, while mutations in *DA1* and *BIG* BROTHER genes result in larger organs in Arabidopsis (Chen et al., 2021), similar mutations in maize do not produce growth-related phenotypes (Gong et al., 2022b), despite gene conservation. However, we cannot exclude the possibility that the roles of Arabidopsis TFs in the stresses examined here remain unexplored, as negative findings are often unreported due to publication bias against non-positive results (Nimpf and Keays, 2020). Fortunately, there is a growing acknowledgment of the value of reporting such “lost in translation” findings, as evidenced by recent publications (Gong et al., 2022b). The extensive availability of public data across diverse stresses and species presents a unique opportunity to accurately identify genes that confer stress resilience (Julca et al., 2023), emphasizing the importance of cross-species analyses and the potential for translational insights into stress tolerance mechanisms.

To maximize the impact of these findings, it is important to consider their potential applications in crop improvement and smart agriculture. The conserved stress-responsive genes and regulatory modules identified here serve as promising candidates for genetic engineering or marker-assisted selection to enhance nutrient stress tolerance in leafy crops. Given the relevance of controlled-environment agriculture, these targets may support the development of high-yield cultivars optimized for indoor or vertical farming systems. Additionally, the stress treatment and transcriptomic analysis framework used in this study—standardized across multiple species and stress conditions—is broadly applicable to other leafy crops. While currently limited to leaf tissues and transcriptomic data, the platform can be extended to incorporate root responses and additional omics layers. To support further research and data exploration, we developed the StressCoNekT database (https://stress.plant.tools), which enables users to query conserved gene expression profiles and predicted gene regulatory networks. The tool is especially useful for researchers and breeders working with leafy vegetables in hydroponic or controlled environments, though users should note that it presently excludes root data and post-transcriptional regulation. Future iterations will aim to address these limitations.

## Data Availability

The raw sequencing data is available at ENA under the accession number: E-MTAB-14018.

## Supplementary material

Supplementary Data Sheet 1 Expression matrices of cai xin.

Supplementary Data Sheet 2 Expression matrices of lettuce.

Supplementary Data Sheet 3 Expression matrices of spinach.

Supplementary Data Sheet 4 Gene regulatory networks for cai xin, lettuce and spinach.
