# Supplementary material for: Cross-species analysis of abiotic stress in hydroponic leafy crops reveals conserved regulatory networks and key divergences
Source: Front Plant Sci. 2025 Jul 7;16:1613016. doi: 10.3389/fpls.2025.1613016 (PMC12277361; doi:10.3389/fpls.2025.1613016)
Supplement: Supplementary Data Sheet 3 — Expression matrices of spinach. [file DataSheet3.pdf]

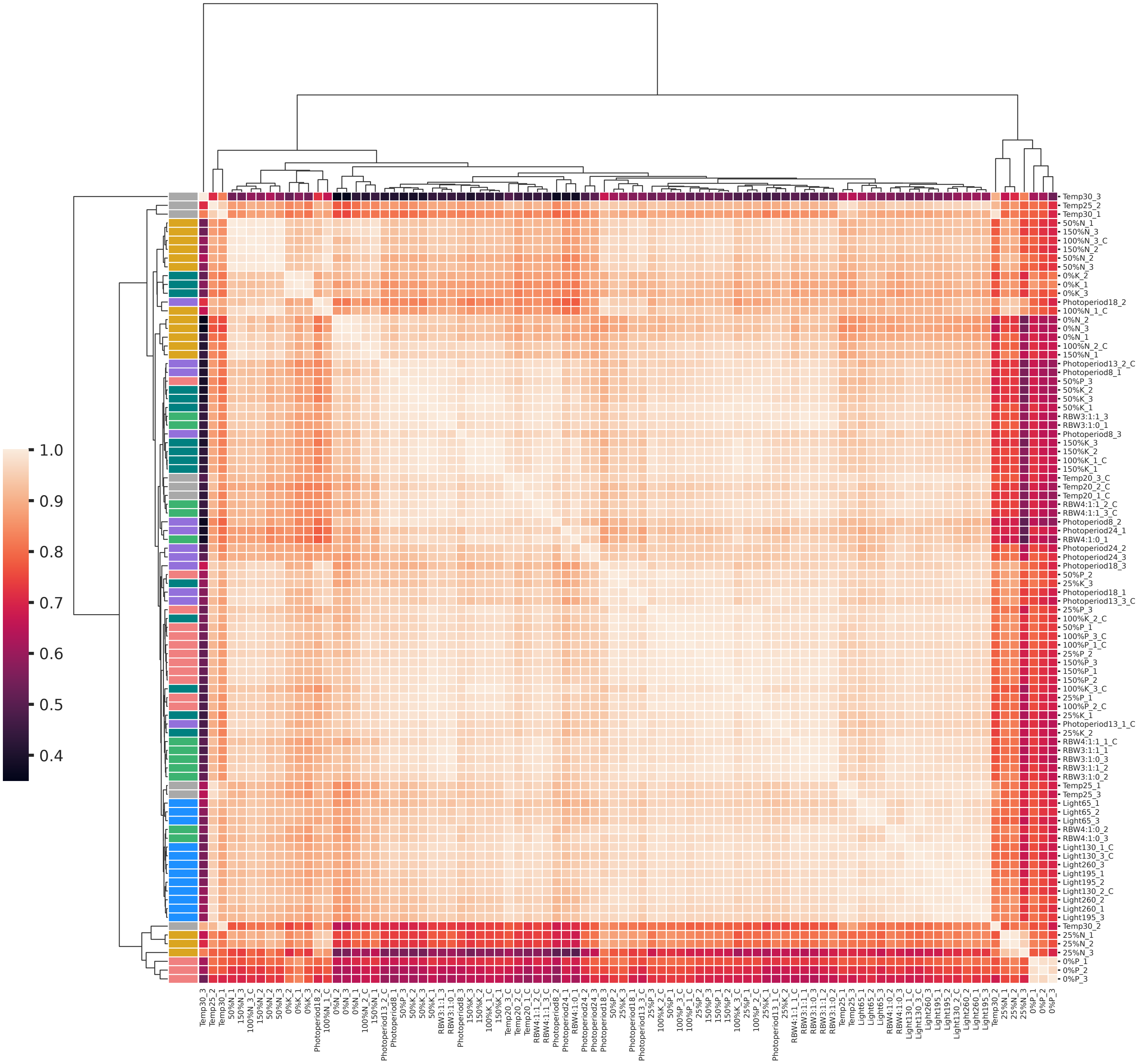

Figure S3. Cluster map of spinach. Pearson correlation coefficient between all the samples of spinach based on the TPM gene expression values. Hierarchical clustering of the samples indicates similarity between the samples and stress conditions.
