# Supplementary material for: Cross-species analysis of abiotic stress in hydroponic leafy crops reveals conserved regulatory networks and key divergences
Source: Front Plant Sci. 2025 Jul 7;16:1613016. doi: 10.3389/fpls.2025.1613016 (PMC12277361; doi:10.3389/fpls.2025.1613016)
Supplement: Supplementary Data Sheet 4 — Gene regulatory networks for cai xin, lettuce and spinach. [file DataSheet4.pdf]

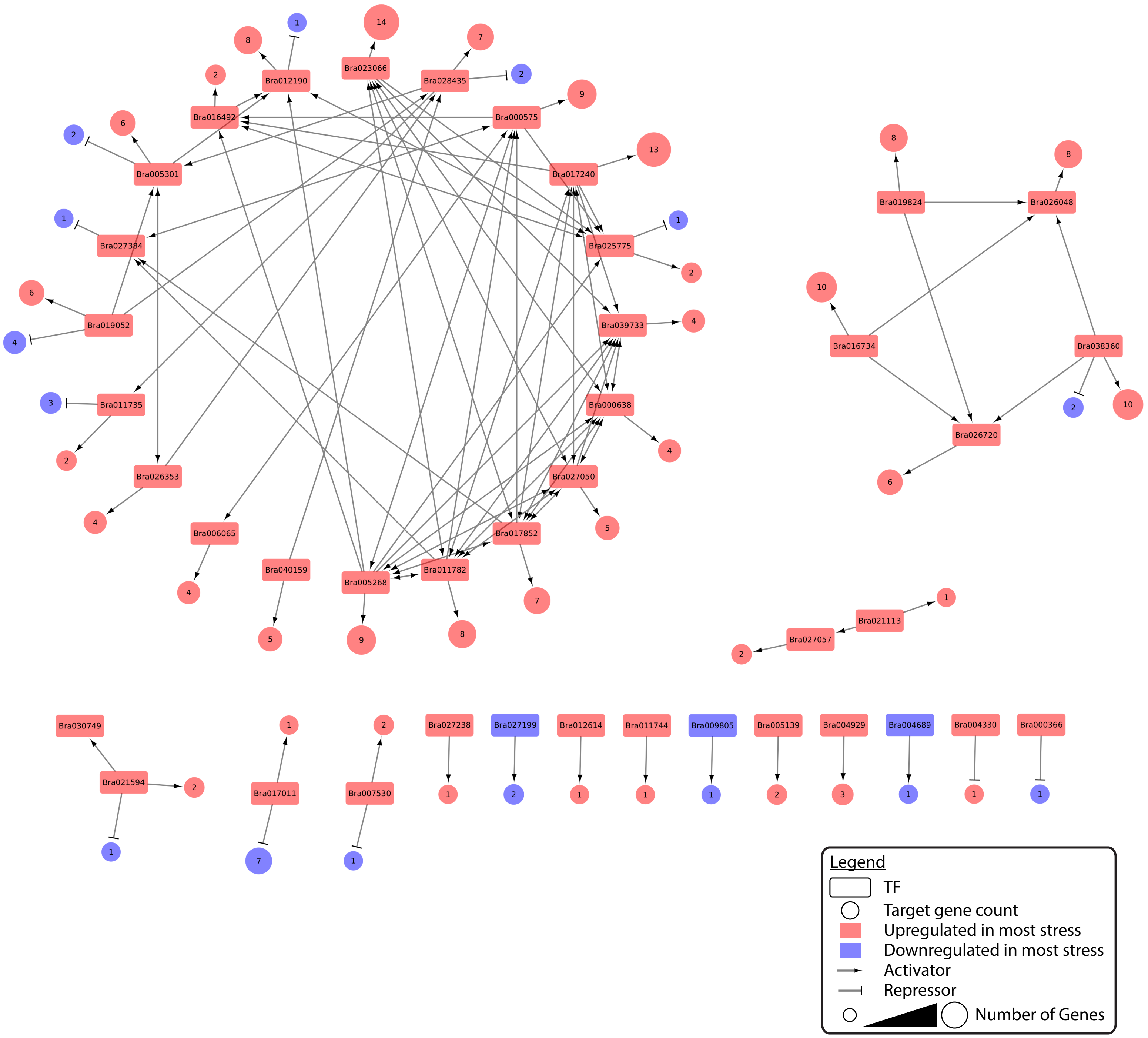

Figure S4. Conserved gene regulatory network in cai xin under phosphate deficiency. Transcription factors (TFs) and target gene pairs are conserved in at least two species under same stress conditions and expression profile (up- or down-regul;ated).. The TFs are represented by rectangular nodes and the size of the circular nodes is indicative of the number of target genes a TF regulates, where larger sizes represent a larger group of genes. Delta and T arrows indicate activators and repressors, respectively. Red color represents upregulation in the conserved stress condition-specific GRN, and blue represents downregulation.
