# Supplementary material for: Cross-species analysis of abiotic stress in hydroponic leafy crops reveals conserved regulatory networks and key divergences
Source: Front Plant Sci. 2025 Jul 7;16:1613016. doi: 10.3389/fpls.2025.1613016 (PMC12277361; doi:10.3389/fpls.2025.1613016)
Supplement: Supplementary file 5 [file DataSheet5.pdf]

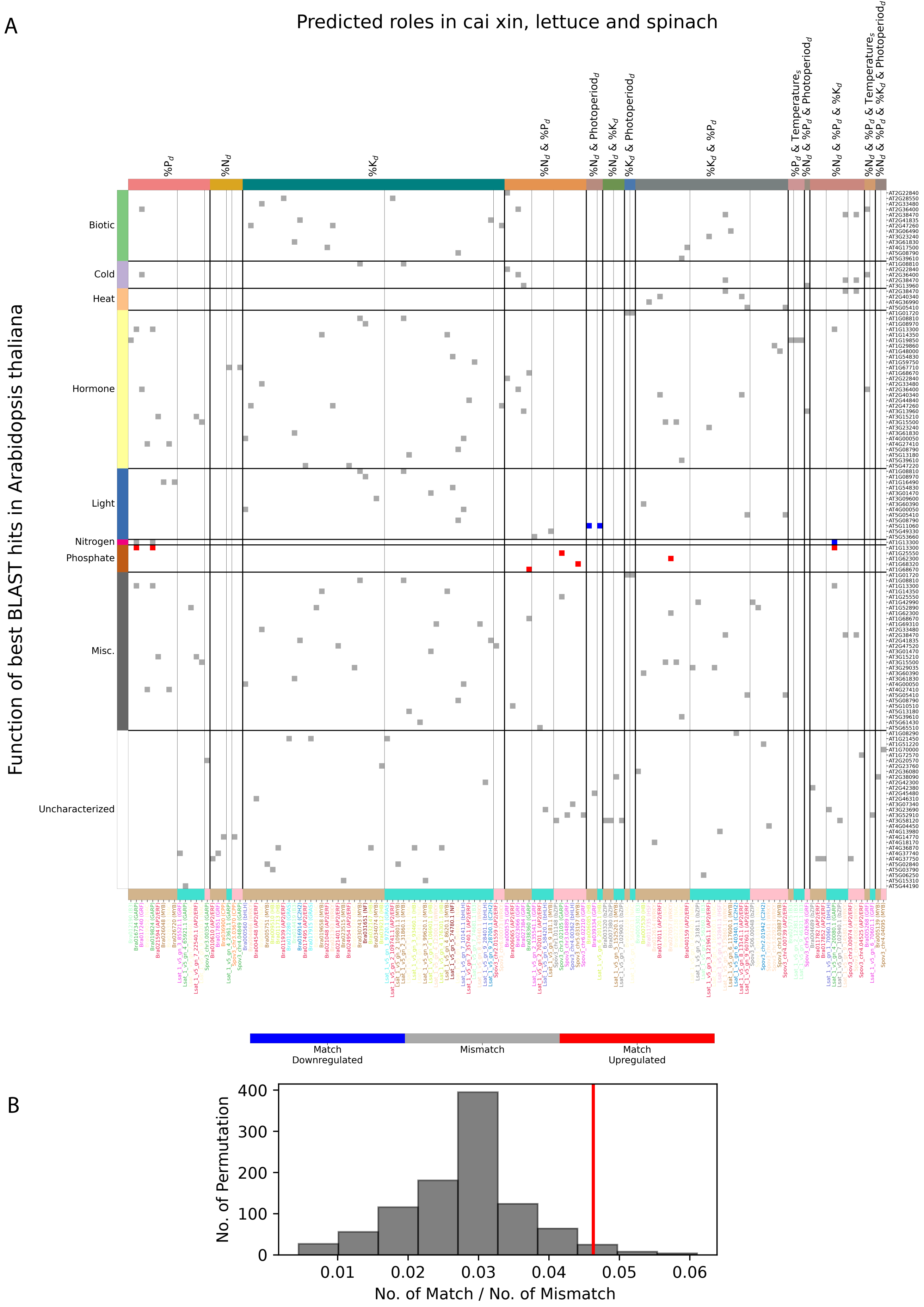

Figure S5. Comparison of stress-responsive transcription factors in lettuce, spinach and cai xin to corresponding experimentally verified genes in *Arabidopsis thaliana*. A) The heatmap indicates the conserved transcription factors (columns) of cai xin (tan), lettuce (turquoise) and spinach (pink), and their corresponding Arabidopsis thaliana best BLAST hits (rows). The genes are grouped by the stress conditions at which the TFs were differentially expressed, and the different stress conditions and combinations are color-coded (top column colors). The stress conditions include P, N, K, photoperiod deficiencies (subscript d), and surplus (subscript s) temperature. The corresponding best hits are grouped according to their functions (row colors) obtained from research papers. Red and blue cells indicate cases where the reported function of Arabidopsis TF corresponds to the same predicted role in our experiments (matches), while gray squares indicate mismatches. B) The observed ratio of matches to mismatches (red line) to the permuted ratio of matches to mismatches (gray histogram).
